# Chemical–biological characterization of a cruzain inhibitor reveals a second target and a mammalian off-target

**DOI:** 10.3762/bjoc.9.3

**Published:** 2013-01-04

**Authors:** Jonathan W Choy, Clifford Bryant, Claudia M Calvet, Patricia S Doyle, Shamila S Gunatilleke, Siegfried S F Leung, Kenny K H Ang, Steven Chen, Jiri Gut, Juan A Oses-Prieto, Jonathan B Johnston, Michelle R Arkin, Alma L Burlingame, Jack Taunton, Matthew P Jacobson, James M McKerrow, Larissa M Podust, Adam R Renslo

**Affiliations:** 1 Small Molecule Discovery Center, University of California San Francisco, 1700 4th Street, San Francisco, CA, 94158, USA; 2 Department of Pharmaceutical Chemistry, University of California San Francisco, 1700 4th Street, San Francisco, CA, 94158, USA; 3 Department of Cellular and Molecular Pharmacology, University of California San Francisco, 1700 4th Street, San Francisco, CA, 94158, USA; 4 Center for Discovery and Innovation in Parasitic Diseases, University of California San Francisco, 1700 4th Street, San Francisco, CA, 94158, USA; 5 Department of Pathology, University of California San Francisco, 1700 4th Street, San Francisco, CA, 94158, USA; 6 Cellular Ultra-Structure Laboratory, Oswaldo Cruz Institute (IOC), FIOCRUZ, Rio de Janeiro, RJ, Brazil 21040-362

**Keywords:** activity-based probes, Chagas’ disease, cruzain, CYP51, 14-α-demethylase, hybrid drugs, *Trypanosoma cruzi*

## Abstract

Inhibition of the *Trypanosoma cruzi* cysteine protease cruzain has been proposed as a therapeutic approach for the treatment of Chagas’ disease. Among the best-studied cruzain inhibitors to date is the vinylsulfone K777 (**1**), which has proven effective in animal models of Chagas’ disease. Recent structure–activity studies aimed at addressing potential liabilities of **1** have now produced analogues such as *N*-[(2*S*)-1-[[(*E*,3*S*)-1-(benzenesulfonyl)-5-phenylpent-1-en-3-yl]amino]-3-(4-methylphenyl)-1-oxopropan-2-yl]pyridine-4-carboxamide (**4**), which is trypanocidal at ten-fold lower concentrations than for **1**. We now find that the trypanocidal activity of **4** derives primarily from the inhibition of *T. cruzi* 14-α-demethylase (*Tc*CYP51), a cytochrome P450 enzyme involved in the biosynthesis of ergosterol in the parasite. Compound **4** also inhibits mammalian CYP isoforms but is trypanocidal at concentrations below those required to significantly inhibit mammalian CYPs in vitro. A chemical-proteomics approach employing an activity-based probe derived from **1** was used to identify mammalian cathepsin B as a potentially important off-target of **1** and **4**. Computational docking studies and the evaluation of truncated analogues of **4** reveal structural determinants for *Tc*CYP51 binding, information that will be useful in further optimization of this new class of inhibitors.

## Introduction

The kinetoplastid protozoan *Trypanosoma cruzi* is the causative agent of Chagas’ disease, a leading cause of heart failure in endemic regions of Latin America [1]. The parasite is transmitted by the reduviid bug and the disease manifests in an initial acute phase, followed by a chronic phase that can last decades and typically culminates in heart failure. The existing treatment for Chagas’ disease involves extended therapy with nifurtimox or benznidazole, both of which are associated with undesirable side-effects and have limited efficacy against the chronic stage of the disease [2–3]. This situation has spurred the search for more effective and better tolerated therapeutics [4–6]. Among a number of drug targets being investigated are cruzain [7–10], the major cysteine protease active in the parasite, and *T. cruzi* CYP51 (*Tc*CYP51), a 14-α-demethylase enzyme of the cytochrome P450 family required for ergosterol biosynthesis [11–14]. *Tc*CYP51 is analogous to the fungal enzyme targeted by the azole class of antifungals, and the observation that some of these drugs (e.g., posaconazole) also inhibit *Tc*CYP51 has led to their preclinical and clinical investigation as potential new treatments for Chagas’ disease [2,15–16].

**Figure 1:**
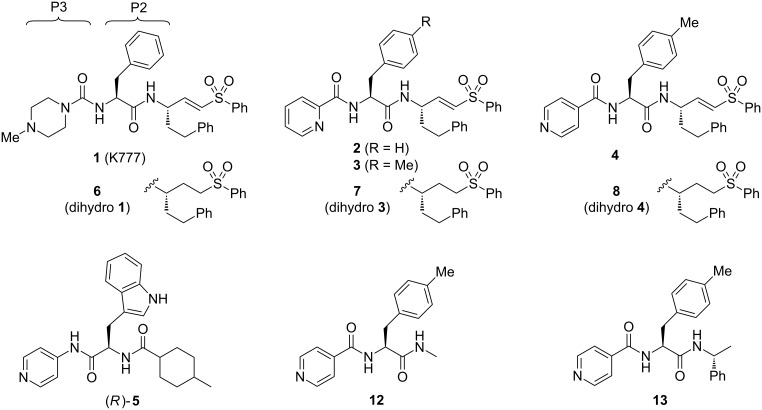
Chemical structures of vinylsulfone-based cruzain inhibitors **1**–**4**, known *Tc*CYP51 inhibitor **5**, dihydro controls **6**–**8**, and truncated analogues **12** and **13**. The “P2” and “P3” side chains of **1** are labeled and bind, respectively, in the S2 and S3 sub-sites of the cruzain active site.

Cruzain is a cathepsin-L-like protease of the papain family thought to be important for intracellular replication and differentiation of the *T. cruzi* parasite [17]. A variety of small-molecule cruzain inhibitors have been described, the majority of which act irreversibly by reaction with the catalytic cysteine in the enzyme active site [18–27]. One of the earliest cruzain inhibitors identified and perhaps the best studied to date is the vinysulfone K777 (**1**, Figure 1). This irreversible inhibitor has demonstrated efficacy in animal models of Chagas’ disease [28–29] and continues to undergo preclinical evaluation leading towards a possible human clinical trial.

Despite many favorable properties, some aspects of **1** are suboptimal from a drug-development perspective. For example, compound **1** is known to be a mechanism-based (irreversible) inhibitor of CYP3A4, an enzyme responsible for the metabolism of many drugs, including **1** itself [30]. In pharmacokinetic studies, compound **1** exhibits nonlinear exposure with escalating dose and is known to be a substrate of the drug transporter P-glycoprotein (P-gp). Finally, as a basic (protonatable) drug species, **1** could potentially accumulate in acidic lysosomes, where mammalian cathepsins (potential off-targets of **1**) are located. The issue of lysosomotropism figured prominently in the discovery and clinical development of cathepsin K inhibitors for osteoporosis. The first such inhibitor to successfully navigate human clinical trials is odanacatib, which was intentionally designed as a nonbasic drug species to minimize the potential for lysosomotropic behavior [31–32].

We sought to address the question of lysosomotropism by preparing analogues of **1** in which the basic piperazine substituent at “P3” (which binds the S3 subsite of cruzain) was replaced with nonbasic or weakly basic heterocycles. In our initial structure–activity study [21], we found that analogue **2** (Figure 1), bearing a 2-pyridylamide at the P3 position, possessed trypanocidal activity that was on par with **1** (Table 1). However, none of the nonbasic analogues examined proved superior to **1** and only 2-pyridyl analogues such as **2** and **3** appeared even comparable. We therefore turned to more dramatic structural alteration and successfully identified and structurally characterized a new nonpeptidic cruzain inhibitor chemotype [24]. Most recently, we returned to reinvestigate nonbasic analogues of **1** and now report that 4-pyridyl analogues such as **4** (Figure 1) are significantly more trypanocidal than **1** or **2**, and unexpectedly exert their trypanocidal effects primarily by inhibition of *Tc*CYP51 rather than cruzain.

## Results and Discussion

### Structure–activity studies

Our exploration of the P3 position in **1** included the evaluation of regioisomeric 2-, 3-, and 4-pyridyl congeners in the context of various P2 side chains. In many such analogue series, we found that regioisomeric analogues possessed similar cruzain activities in vitro, while the 4-pyridyl examples consistently demonstrated superior trypanocidal activity against cultured *T. cruzi* parasites. For example, 4-pyridyl analogues (e.g., **4**) exhibited sub-micromolar minimal trypanocidal concentration values (MTC = 0.6 μM) while the MTC values for 2-pyridyl (e.g., **3**) and 3-pyridyl analogues were typically ≈10 μM, which was similar to the MTC of **1** (Table 1). The MTC represents the minimum concentration of test compound required to completely clear *T. cruzi* parasites from J774 macrophage host cells over a 40-day experiment, with the test compound being administered during the initial 28 days.

**Table 1:** In vitro biochemical and cellular activities of test compounds and controls. (n.a. = not active (cruzain IC_50_ > 50 μM); BNZ = benzindazole; POSA = posaconazole).

compound	cruzain activity	*Tc*CYP51 activity		*T. cruzi* growth inhibition	
	*k*_inact_/*K*_i_ (s^−1^·M^−1^)	in vitro *K*_D_ (nM)	cellular activity (Y/N, conc.)^a^	MTC^b^ (μM)	HCS^c^ EC_90_ (μM)

**1**	118,000	>2,000	N (1.6 μM)	8	0.10
**2**	120,000	—	—	10	—
**3**	16,000	>2,000	N (2.0 μM)	8	1.85
**4**	67,300	≤5	Y (0.2 μM)	0.6	0.10
**5**	—	≤5	Y (5.0 μM)	≤10^d^	—
**6**	n.a.	>2,000	N (2.0 μM)	>10	>10
**7**	n.a.	>2,000	N (0.1 μM)	>10	>10
**8**	n.a.	≤5	Y (0.1 μM)	0.25	0.11
**9**	81,500	—	—	5^e^	0.017
**12**	n.a.	620 ± 260	—	>10	>10
**13**	n.a.	75 ± 26	—	1^f^	3.9
BNZ	—	—	—	10	7.2
POSA	—	≤5	Y (0.1 μM)	0.003	2.7

^a^Compound affects ergosterol biosynthesis at indicated concentration as determined by GC/MS analysis. ^b^Minimum effective concentration that clears J774 host cells of parasites at day 40 of the experiment, following 28 days of treatment. ^c^Concentration that reduces parasite load in C2C12 cells by 90% relative to untreated controls. ^d^Concentrations lower than 10 μM were not examined. ^e^Experiment performed in BESM host cell rather than J774 cells. ^f^Read at day 12 following 7 days treatment.

The enhanced potency of 4-pyridyl analogues as compared to **1** or their regioisomeric analogues was not predictable on the basis of in vitro cruzain activity (Table 1). Nor could the trends be explained as an effect of lysosomotropism, since enhanced potency was observed only for the 4-pyridyl analogues and not for 2- or 3-pyridyl analogues, which have similar p*K*_a_ values. Instead, we considered that additional target(s) may be responsible for the surprising potency of the 4-pyridyl analogues. Specifically, we were aware that a 4-pyridyl ring comprises the putative heme-binding moiety in a new class of *Tc*CYP51 inhibitors represented by compound **5** (Figure 1). Other structural similarities of **4** and **5** suggested that compound **4** could conceivably bind *Tc*CYP51.

To test the hypothesis that **4** may also target *Tc*CYP51, we examined the binding of this compound to *Tc*CYP51 using a UV–vis spectroscopic binding assay described previously [33]. Indeed, compound **4** bound *Tc*CYP51 with an estimated *K*_D_ ≤ 5 nM, a value comparable to the binding affinity of the known *Tc*CYP51 inhibitor **5** [16]. 2-Pyridyl analogue **3** did not measurably bind *Tc*CYP51 (*K*_D_ > 2,000 nM, Table 1), whereas the corresponding 3-pyridyl congener (not shown) binds about 100-fold more weakly (*K*_D_ ≈ 500 nM) than **4**. These findings were thus consistent with our hypothesis that the 4-pyridyl ring in **4** is involved in binding *Tc*CYP51. The 2-pyridyl ring system in **3** is presumably unable to chelate heme in *Tc*CYP51 due to steric hindrance from the immediately adjacent amide linkage.

### Computational docking studies

**Figure 2:**
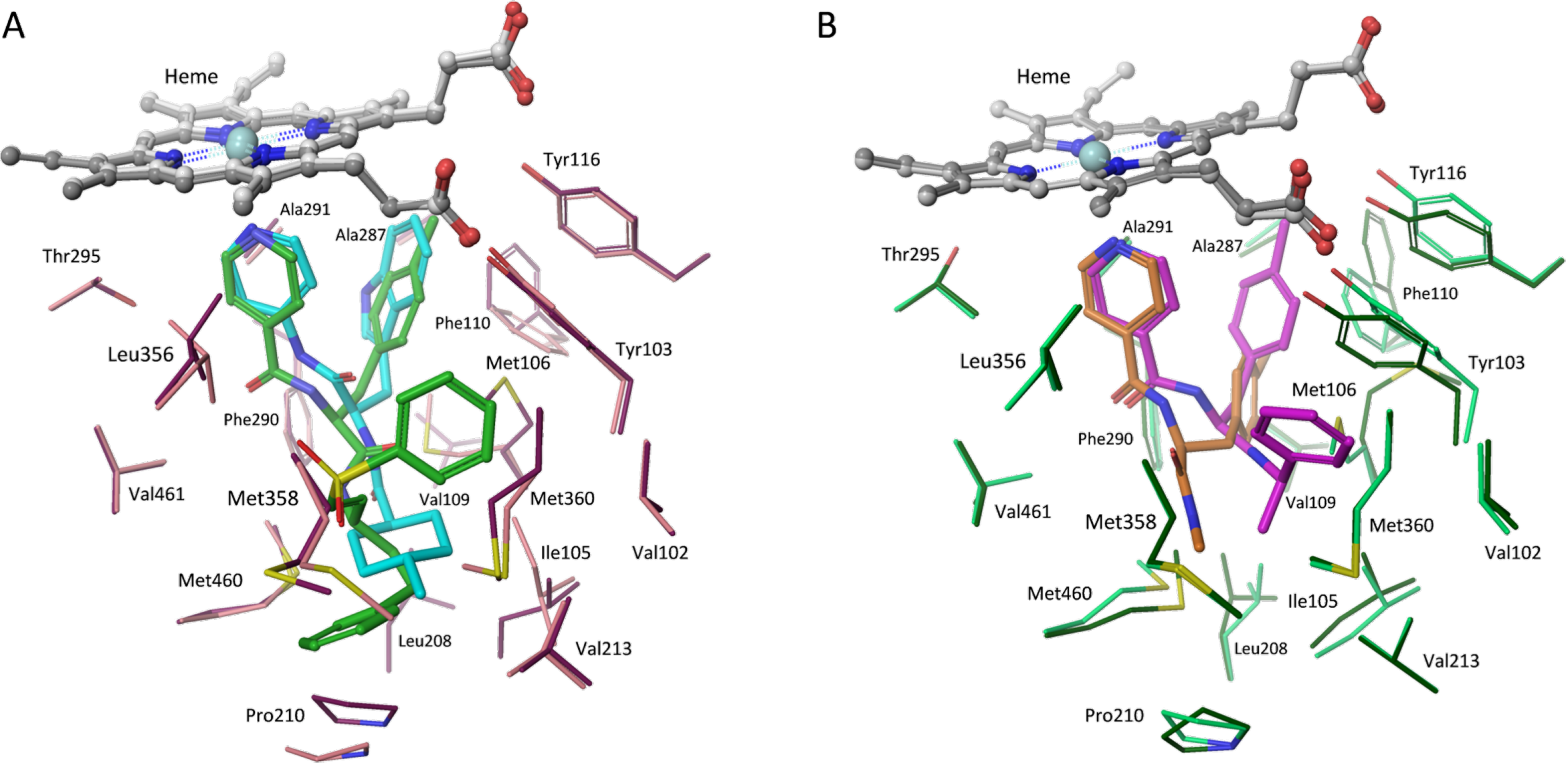
Computational docking models. (A) Predicted binding modes of **4** and (*R*)-**5** bound to *Tc*CYP51. For **4**, the ligand, the protein, and the heme group are shown in green, pink, and grey, respectively. For (*R*)-**5**, the ligand, the protein, and the heme group are shown in cyan, purple, and white, respectively. Heme-iron chelation and hydrophobic binding interactions dominate in the models. (B) Predicted binding models of truncated analogues **12** and **13** to *Tc*CYP51. For **12**, the ligand, the protein, and the heme group are shown in orange, light green, and light grey, respectively. For **13**, the ligand, the protein, and the heme group are shown in magenta, dark green, and dark grey, respectively.

We next employed computational docking and a model derived from the crystal structure of *Tc*CYP51 to compare predicted binding modes of **4** and (*R*)-**5**. The two ligands were docked by using the induced-fit docking protocol with Glide XP [34], and the models were further refined by minimizing the energies of the ligand and surrounding residues (within 5 Å of ligand) using PRIME [35]. Finally, binding scores were computed by using both Glide XP and the MM/GMSA method. Compound **4** was predicted to bind in a similar fashion as (*R*)-**5**, with the 4-pyridyl ring chelating the heme-iron atom and the tolyl ring at P2 contacting many of the same residues (e.g., Try103, Phe110) predicted to interact with the tryptophan ring of (*R*)-**5** (Figure 2A). This same hydrophobic site in *Tc*CYP51 binds the fluoroaryl rings of fluconazole and posaconazole in co-crystal structures [14]. The predicted binding mode of the enantiomer (*S*)-**5** was described previously [16] and is distinct from that proposed for **4** and (*R*)-**5**.

Thus, computational docking provides a conceptual picture of how compound **4** – notionally a cruzain inhibitor – might also bind *Tc*CYP51. Interestingly, this is not the first time that potent *Tc*CYP51 binding has been discovered in a molecule originally intended for a different target. Buckner and Gelb unexpectedly found that the human protein farnesyltransferase (PFT) inhibitor tipifarnib exerts its antitrypanosomal effects through inhibition of *Tc*CYP51 [36]. Subsequently, these researchers succeeded in divorcing PFT activity from *Tc*CYP51 inhibition in the tipifarnib scaffold, producing new lead compounds with compelling properties [37–39].

### Inhibition of mammalian CYPs

A concern with any inhibitor of *Tc*CYP51 is the potential for cross reactivity with mammalian cytochrome P450 (CYP) enzymes, especially those CYPs involved in drug metabolism, like CYP3A4. To assess this risk, we evaluated the inhibitory activities of **4** and **1** across a panel of relevant mammalian CYP enzymes (Table 2). Both **4** and **1** inhibited all CYPs in the panel, with IC_50_ values generally in the low micromolar range. Although compound **4** did inhibit CYP3A4, the potency of inhibition (IC_50_ = 0.8 μM) was less than that exhibited by the antifungal drug ketoconazole (IC_50_ = 0.086 μM). It should be noted that the substrate-derived IC_50_ values from the CYP panel are not directly comparable to the *K*_D_ values for binding to *Tc*CYP51. What can be said is that the antitrypanosomal effects of **4** are realized at concentrations (EC_90_ = 0.1 μM, MTC = 0.5 μM) well below the in vitro potency of the compound across the CYP panel (average IC_50_ ≈ 7 μM). Compound **4** thus possesses reasonable selectivity with regard to off-target CYP inhibition, and represents a reasonable starting point from which further improvements in selectivity may be undertaken.

**Table 2:** In vitro inhibition of important mammalian CYP enzymes.

compound	IC_50_ (μM)				
	1A2	2C9	2C19	2D6	3A4

**1**	24	32	7.6	26	1.7
**4**	22	5.5	3.4	2.7	0.8
ketoconazole	—	—	—	—	0.086

Given the similar IC_50_ values for **1** and **4** against CYP3A4, we were curious to determine whether **4** is an irreversible inhibitor of this enzyme, as is the case for **1** [30]. Irreversible inhibition is typically assessed by measuring the activity of microsomal CYPs following pre-incubation with or without NADPH. Consistent with earlier studies [30], compound **1** exhibited irreversible inhibition of CYP3A4 as reflected in a significantly lower IC_50_ value with NADPH pre-incubation (Table 3). In contrast, compound **4** showed behavior typical of reversible inhibition, with no NADPH-dependent shift in the IC_50_ value. In the case of CYP2C19, both compounds were found to be reversible inhibitors. These results suggest that CYP inhibition by **4** involves reversible binding of the parent molecule, while the inhibition of CYP3A4 conferred by **1** is dependent on initial conversion to a reactive metabolite. Whatever the explanation, reversible inhibition of CYP enzymes (as with **4**) is clearly preferable to irreversible inhibition from a drug-safety perspective.

**Table 3:** Mechanism of inhibition studies for compounds **1** and **4**. These data suggest that inhibition of CYP3A4 by **1** is irreversible in nature.

compound	2C19 IC_50_ (μM)		3A4 IC_50_ (μM)	
	+NADPH	−NADPH	+NADPH	−NADPH

**1**	5.54	8.22	**0.0059**	1.08
**4**	0.300	0.170	0.117	0.046

### Inhibition of *Tc*CYP51 in live parasites

**Figure 3:**
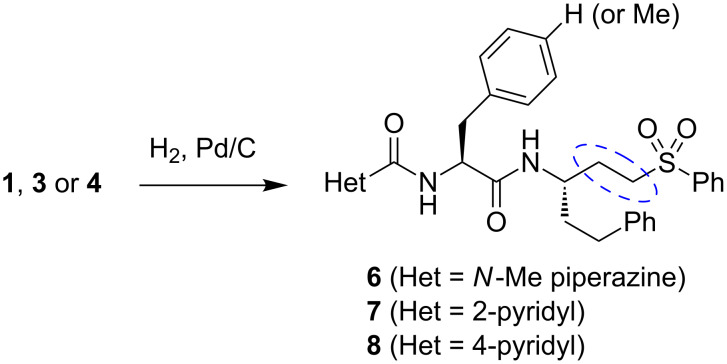
Synthesis of the additional control compound **6**–**8**, the reduced forms of analogues **1**, **3**, and **4** respectively.

We next sought to better define the relative importance of *Tc*CYP51 and cruzain inhibition in the antitrypanosomal effects of compound **4**. Since the 2-pyridyl analogue **3** was found to not bind *Tc*CYP51, this compound could serve as a control for the cruzain-derived (and/or other cysteine-protease-derived) effects of **4**. To provide controls lacking activity against cysteine proteases, we reduced the vinylsulfone function in analogues **1**, **3**, and **4** to afford the dihydro analogues **6**–**8** (Figure 3). As expected, these analogues were devoid of any detectable cruzain inhibitory activity (IC_50_ > 50 μM, Table 1). Compounds **3**, **4**, **7** and **8** thus comprised a set of analogues with complementary activity profiles against the two putative targets: **4** (cruzain and *Tc*CYP51 inhibition), **8** (*Tc*CYP51 inhibition only), **3** (cruzain inhibition only), and **7** (neither activity).

Compounds **3**, **4**, **7**, and **8** were evaluated for potency against intracellular *T. cruzi* parasites by using two different assays. The reported EC_90_ values (Table 1) represent compound concentrations required to reduce parasite numbers in C2C12 host cells by 90% as compared to untreated controls, as determined by using a high-content imaging-based screening (HCS) approach [33,40]. This high-throughput assay provides a rapid measure of the initial acute effects of test compound on parasite viability. The more laborious MTC assay identifies compound concentrations that clear parasites from the host cell, as determined ca. two weeks after the conclusion of a four-week course of treatment. This MTC assay therefore provides a measure of trypanocidal action that cannot be drawn from the more rapid HCS assay. We judge that MTC values are more representative of the therapeutic drug levels that would likely be required to produce efficacy in an animal model of Chagas’ disease.

The antitrypanosomal effects of compounds **3**, **4**, **7**, and **8** were in general agreement with their in vitro activities against the two putative targets (Table 1). Analogue **7**, devoid of either activity in vitro*,* showed no effects on *T. cruzi* parasites in either the HCS or MTC assay. Analogue **3**, possessing primarily cysteine-protease-derived effects, was effective in both assays and equipotent to **1** in the MTC assay. Putatively dual-targeted analogue **4** was about 10-fold more potent than **1** in the MTC assay and equipotent by HCS. Most unexpectedly, we found that compound **8**, which lacks any cruzain-derived effects of **4**, was equipotent to **4** by HCS and 2–4 times more potent than **4** in the MTC assay.

**Figure 4:**
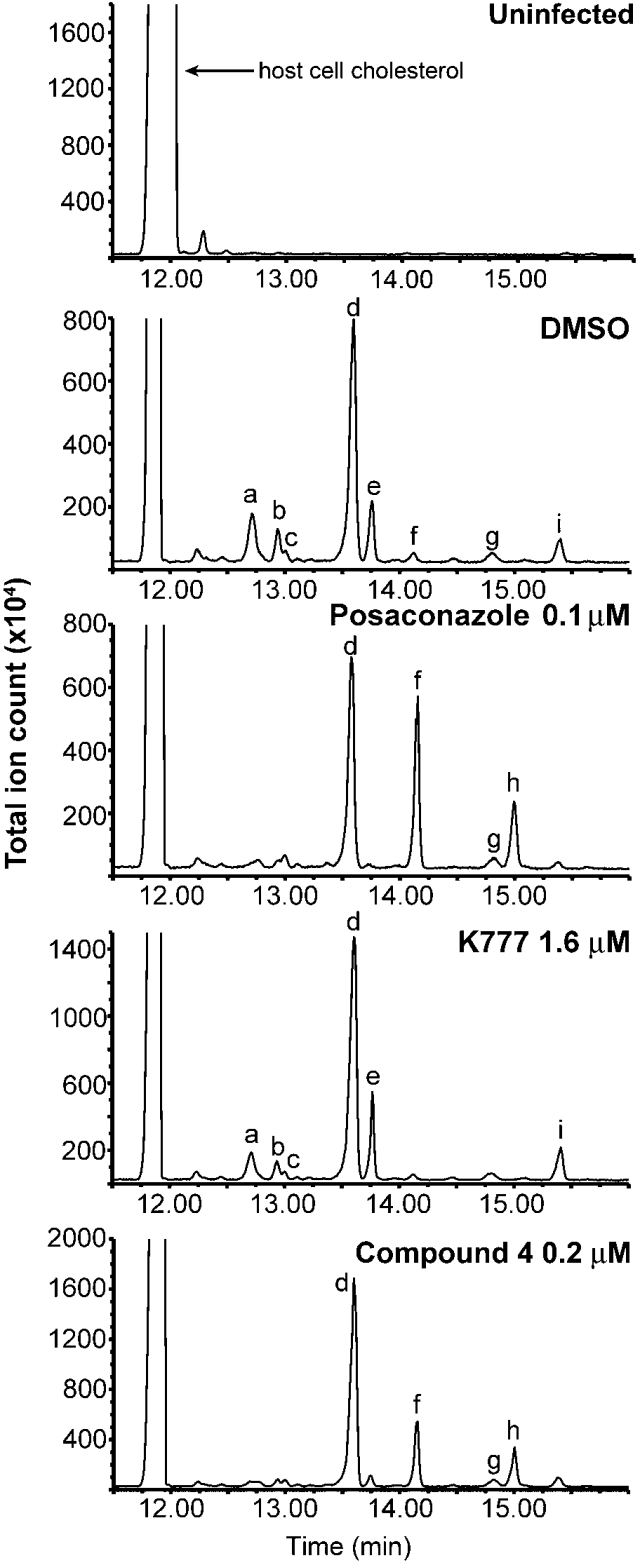
GC/MS analysis of lipid extracts from *T. cruzi* parasites treated with test compounds. DMSO and K777 (**1**) were used as negative controls; posaconazole served as a positive control. The analysis of **4** was performed concurrently with other CYP51 inhibitors described recently [33] and, thus, the spectra for the controls shown above are reproduced from the earlier report. Spectra of lipid extracts from parasites treated with **3**, **6**, **7**, and **8** are provided in Supporting Information File 1. Uninfected host cell panel (top) demonstrates that chromatographic peaks labeled **a** to **i** in subsequent panels are of *T. cruzi* origin. These peaks are assigned as **a** - cholesta-7,24-dien-3β-ol, [M]^•+^ = *m*/*z* 454; **b** - cholesta-8,24-dien-3β-ol (zymosterol), [M]^•+^ = *m*/*z* 470; **c** - 24-methyl-7-en-cholesta-en-3β-ol, [M]^•+^ = *m*/*z* 472; **d** - ergosta-7,24-diene-3β-ol (episterol), [M]^•+^ = *m*/*z* 470; **e** - ergosta-8,24-diene-3β-ol (fecosterol), [M]^•+^ = *m*/*z* 470; **f** - lanosterol, [M]^•+^ = *m*/*z* 498; **g** - 4-methylepisterol, [M]^•+^ = *m*/*z* 484; **h** - eburicol, [M]^•+^ = *m*/*z* 512; **i** - 24-ethyl-7,24(24’)-encholestadien-3β-ol, [M]^•+^ = *m*/*z* 484.

The in vitro and cell-based activities of **4** and **8** suggest *Tc*CYP51 as a relevant target of these compounds. To assess inhibition of *Tc*CYP51 in live parasites we analyzed the sterol composition of intracellular *T. cruzi* parasites treated with test compounds **3**–**8**, **1**, or posaconazole as a positive control. The analysis was performed by employing GC/MS as reported previously for compound **5** [33]. The GC/MS trace for uninfected host cells establishes that the additional peaks observed in infected cells are of *T. cruzi* origin (peaks labeled **a**-**i**, Figure 4). Treatment with the known *Tc*CYP51 inhibitor posaconazole produces an increase in the relative abundance of *Tc*CYP51 substrates lanosterol (**f**) and eburicol (**h**) and accordingly, a reduction in the abundance of downstream sterols such as fecosterol (**e**) and cholesta-7,24-dien-3β-ol (**a**), among others. Treatment with **1** had little effect on sterol composition as expected, whereas treatment with compound **4** or **8** produced effects very similar to those observed in posaconazole treated parasites (Figure 4 and Supporting Information File 1). The other test compounds evaluated (**3**, **6**, **7**) produced no significant change in lipid composition, as expected since these compounds do not inhibit *Tc*CYP51 in vitro (Supporting Information File 1). Test compounds were necessarily studied at concentrations below their MTC, so as to retain a population of viable parasites for analysis.

### An activity-based probe reveals an off-target of **1** and **4**

**Figure 5:**
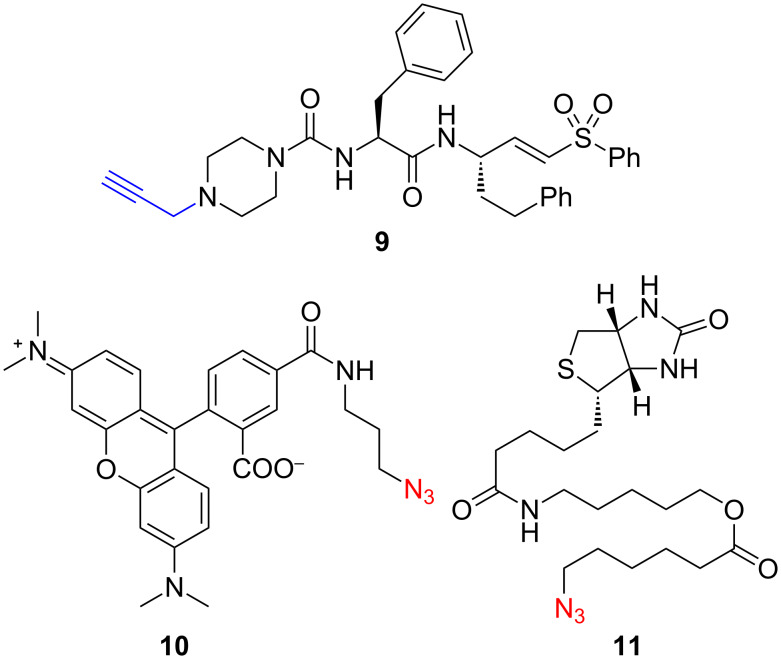
Chemical structures of compound **9**, a “clickable” activity-based probe based on **1** and complementary azide-containing reagents **10** and **11**.

We next sought to evaluate the cysteine-protease-related effects of the various test compounds in *T. cruzi* parasites. To do this, we designed and synthesized the “clickable” activity-based probe **9** in which a propargyl group (replacing methyl in **1**) serves as a chemical handle for conjugation to TAMRA- or biotin-containing reagents (**10** and **11**, respectively, Figure 5). Probe **9** was found to be equipotent to **1** against cruzain in vitro and retained similar effects against *T. cruzi* parasites in both the HCS and MTC assays. Thus, the cysteine protease target(s) of **9** in parasite and host cell can reasonably be assumed to be the same as for **1** and close analogues such as **4**.

Independently, another group recently reported the synthesis of **9** and its use to identify putative targets of **1** in the related parasite *Trypanosoma brucei* [41]. Our efforts to similarly identify targets of **1** in *T. cruzi* were complicated by the presence of a host-cell protein that was apparently a major target of **9**. In a typical experiment, intracellular *T. cruzi* amastigotes were treated with **9** for 1 hour, followed by cell lysis, “click” reaction with TAMRA azide **10**, and separation/visualization by SDS-PAGE. Regardless of the host cell employed (J774 macrophage, or C2C12), only one prominently labeled band at ≈35 kDa was observed in these experiments. This band was attributed to a host-cell protein as it appeared also in analogous experiments employing uninfected cells. In fact, we could not conclusively identify any unique bands of parasitic origin in our experiments, although such bands might well have escaped detection due to lower abundance and labeling below the limit of fluorescence detection.

**Figure 6:**
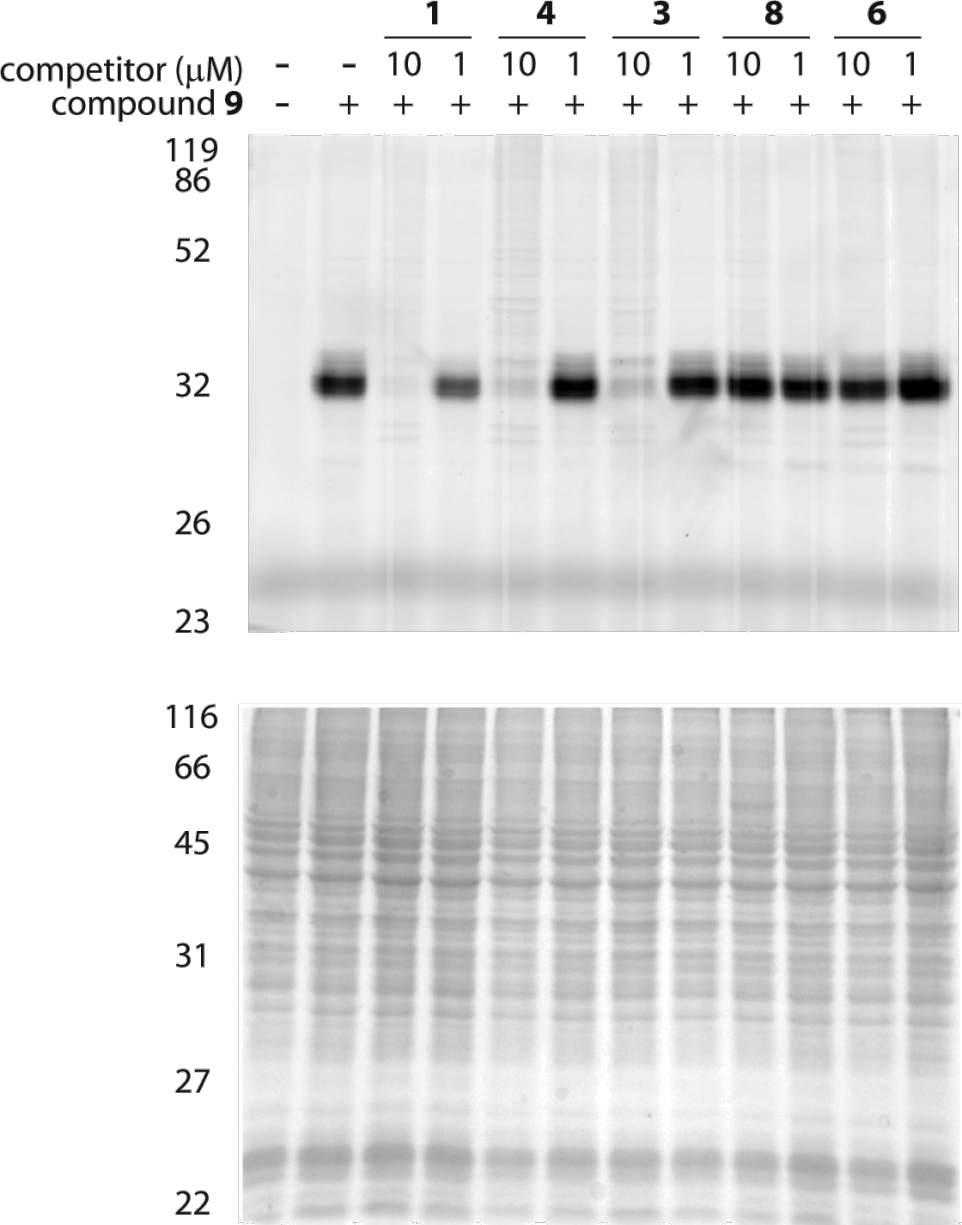
Competitive labeling of host cell (C2C12) proteins. Intact cells were labeled with probe **9** following a competitive pre-incubation step with compounds **1**, **4**, **3**, **8**, or **6**. After cell lysis, protein adducts of **9** were conjugated to rhodamide-based dye **10**. The gel image at the top shows rhodamine fluorescence. The gel image at the bottom is of the coomassie stained gel. A successfully competed band is observed at ≈35 kDa, and this was subsequently identified as cathepsin B.

The discovery of a potential mammalian off-target of probe **9** (and presumably also of **1**) was of considerable interest, so we explored this finding further. To determine if this protein was also a target of **1** and **4**, we conducted competition experiments in C2C12 cells. Hence, pre-incubation of cells with competitor compound at either 1 μM or 10 μM for one hour was followed by treatment for one hour with **9**, followed by cell lysis, conjugation to **10**, separation (SDS-PAGE), and detection by rhodamine fluorescence as before. In these experiments, pretreatment with 10 μM of compound **1**, **3**, or **4** successfully blocked labeling of the ≈35 kDa band by probe **9**, thus indicating that these compounds also react with this target (Figure 6). As expected, the nonelectrophilic dihydro forms of **1** and **4** (i.e., compounds **6** and **8**) did not compete for labeling by **9**. Taken together, these results strongly suggest that compounds **1**, **3** and **4** react irreversibly with the ≈35 kDa protein in a process involving the electrophilic vinylsulfone moiety.

### Chemical proteomics

**Figure 7:**
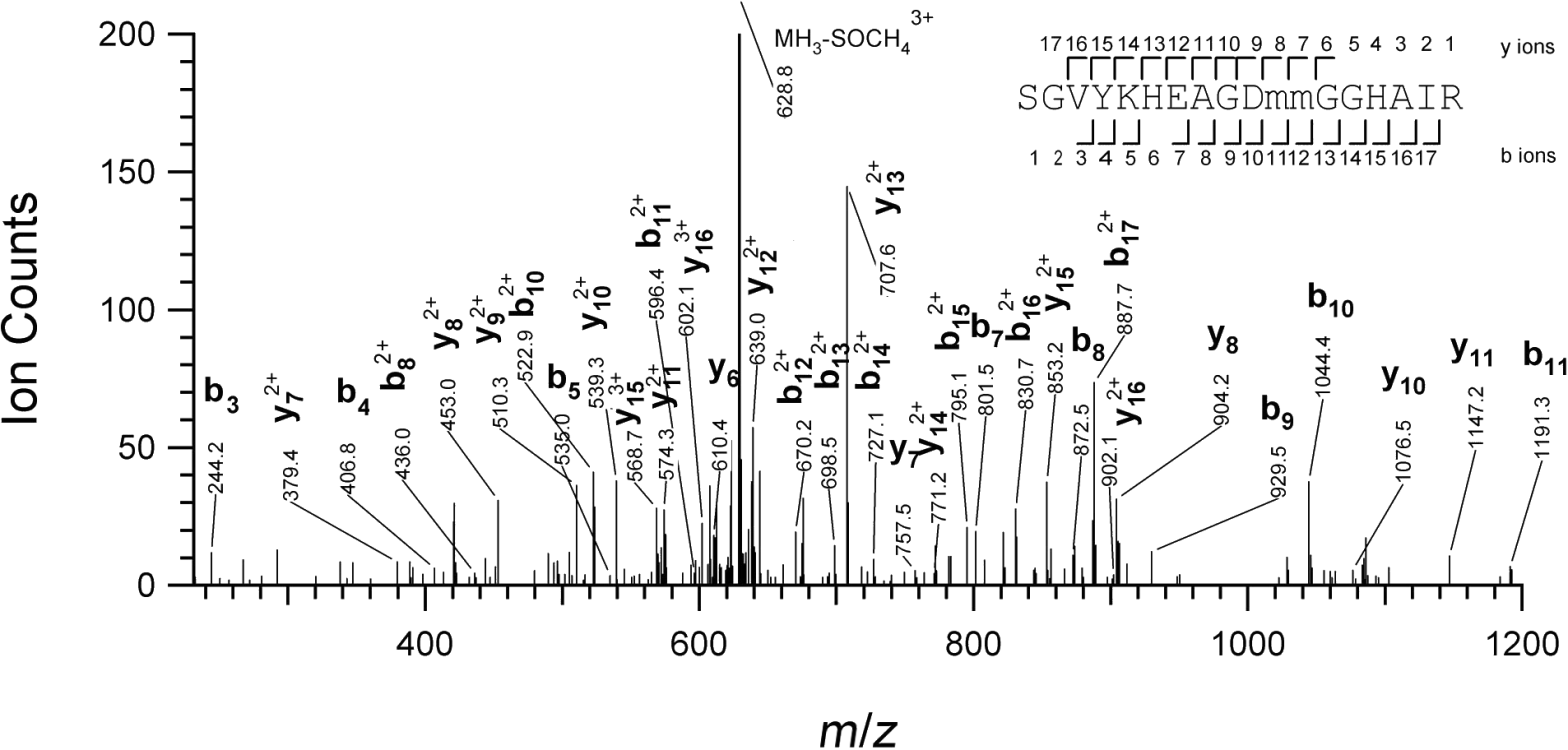
MS/MS spectrum of the tryptic peptide S264-R281 from mouse cathepsin B, identified in pull-down experiments employing compound **9** in C2C12 cells. Observed sequence ions are labeled (m = oxidized methionine).

We next applied mass spectrometric analysis to identify the ≈35 kDa band, which was an apparent target of the electrophilic inhibitors described above. To enrich for this protein, C2C12 cells were labeled with **9** as before and then reacted with the biotin azide reagent **11**, followed by biotin capture onto streptavidin beads. A base-cleavable ester function was introduced in the linker of **11**, and this allowed enriched proteins to be released from beads by treatment with sodium hydroxide. The liberated proteins were separated by SDS-PAGE, and the relevant band at ≈35 kDa extracted from the gel. An in-gel trypsin digest [42] was followed by UPLC separation of the tryptic peptides and MS/MS analysis using a hybrid linear ion-trap-Orbitrap mass spectrometer. Tandem mass spectra acquired were searched against the UniProtKb database employing ProteinProspector; four MS/MS spectra corresponding to the same peptide sequence were identified (Figure 7). This sequence was found to correspond to the tryptic peptide spanning resides S264-R281 from mouse cathepsin B (uniprot P10605). Significantly, this peptide was not found in analogous experiments where pre-incubation with **1** or **4** (at 10 μM) preceded labeling with **9**, nor in an experiment in which **9** was not added. Thus, cathepsin B is very likely the host-cell protein target of compounds **1** and **4** identified in the competition experiments with compound **9**.

The identification of cathepsin B as a relevant cellular off-target of **1** and **4** is potentially significant. On the one hand, the HCS EC_90_ values of **1** and **4** are at least 10-fold lower than the concentrations of these compounds used in the competition experiments. Thus, one might expect to achieve effects on parasite viability before significant inhibition of cathepsin B is conferred. On the other hand, the MTC for **1** (8 μM) lies squarely in the range at which the compound effectively competes for cathepsin B labeling by **9**. Thus, if micromolar concentrations of **1** are indeed required to achieve a therapeutic effect in animals, one might well be concerned about the effects on host cathepsin B. Thus, the experiments with **9** identified a potential off-target while also providing an experimental means for testing the effects of new analogues on this off-target in a relevant, cellular context.

### Defining a new lead scaffold for *Tc*CYP51 inhibition

The similar cellular potencies of **4** and its reduced form **8** suggest that cruzain inhibition plays a relatively minor role in the trypanocidal action of **4.** To a first approximation, the cruzain- and *Tc*CYP51-derived effects of **4** should be similar to those of its close analogues **3** (MTC ≈ 10 μM) and **8** (MTC ≈ 0.25 μM), respectively. Unless the effects of inhibiting both targets are synergistic, which is not supported by the data, there would appear to be little benefit gained by combining a relatively weak cruzain-derived effect with a much more potent insult conferred by *Tc*CYP51 inhibition. Moreover, it now seems likely that electrophilic compounds such as **1** and **4** may be partially consumed in nonproductive reactions with host-cell proteases (e.g., cathepsin B) and/or other cytosolic nucleophiles (e.g., glutathione). This possibility is supported by our competitive labeling experiments (Figure 6) and by in vitro studies employing physiological concentrations of glutathione (Supporting Information File 1). With regard to the inhibitor chemotypes covered here, there appears to be little rationale for targeting both cruzain and *Tc*CYP51. On the other hand, the surprising potency of analogue **8** does suggest this as a new lead scaffold for the development of novel *Tc*CYP51 inhibitors.

We next sought to define the minimal pharmacophore within **8** required for inhibition of *Tc*CYP51 in vitro and antitrypanosomal effects in whole cells. We therefore synthesized truncated analogues of **8**, such as **12** and **13** (Figure 1). These compounds retain the 4-pyridyl ring and neighboring tolyl side chain of **8** while dispensing with those substituents further removed from the putative heme-binding moiety. Interestingly, the truncated analogues **12** and **13** bound *Tc*CYP51 significantly more weakly than **4** or **8** (Table 1), suggesting that side chains relatively far removed from the 4-pyridyl ring nonetheless play an important role in binding.

Computational docking of **12** and **13** provided some insight into the observed binding trends. Analogue **13** adopts a docking pose very similar to **4** with respect to the 4-pyridyl and tolyl ring systems. When compared to the poses for **4** or **13**, the tolyl ring in **12** projects much less deeply into the aromatic pocket formed by Phe110 and Tyr103 (Figure 2). Neither **12** nor **13** form interactions with more distal residues (e.g., Leu208, Pro210) that are predicted to form productive contacts with **4**. Thus, a larger number of hydrophobic contacts and better orientation of some side chains may explain the binding trends for **4**, **12**, and **13**. Interestingly, the rank-order binding affinities of **4**, **12**, and **13** were correctly predicted by the MM-GBSA method applied to the binding models of these compounds (Supporting Information File 1). This suggests that such models could serve to aid in the design of new *Tc*CYP51 inhibitors derived from this scaffold.

The antitrypanosomal activities of analogues **12** and **13** could be correlated with their in vitro binding affinities for *Tc*CYP51 (Table 1). Hence, analogue **13** (*K*_D_ = 75 nM) shows reduced antitrypanosomal activity when compared to **8** (*K*_D_ ≈ 5 nM). Still weaker-binding analogue **12** (*K_D_* = 620 nM) exhibited no antitrypanosomal effect at the highest concentration examined (10 μM). Thus, compound **13** can be considered to represent a “minimal pharmacophore” that retains reasonable affinity for *Tc*CYP51 in vitro while also conferring an effect on *T. cruzi* parasites in culture. Future work will focus on further refining the in vitro and cellular potency of this scaffold, with compound **13** serving as a chemical departure point.

## Conclusion

Structure–activity studies are often conducted with the underlying assumption that molecular mechanisms are the same within congeneric analogue series. This assumption is reinforced when activity in biochemical assays can be correlated with cell-based activity. Of course perfect correlation is rarely observed, even when a series is in fact “on-target”. Especially perilous is the construction of mechanistic hypotheses based solely on the correlation of in vitro biochemical assay data with gross phenotypic endpoints such as parasite growth inhibition or cell death. As demonstrated here, even seemingly trivial structural changes within a congeneric SAR series can produce analogues with disparate molecular mechanisms of action. Advisable approaches to deal with these uncertainties include the use of cell-based counter assays that can detect action at specific targets or signaling pathways of interest. Activity-based probes can serve as useful tools to verify on-target action during the course of chemical optimization campaigns.

## Supporting Information

The Supporting Information features a table with experimentally determined and computationally predicted binding affinities, additional GC/MS spectra from lipid-analysis studies, time courses for reaction of compounds **1** and **6** with glutathione in vitro, and synthetic schemes for analogues **4**, **9**, **11**, **12**, and **13**, as well as experimental procedures.

File 1 Figures, schemes, and experimental procedures.
