# Supplementary material for: Chemical–biological characterization of a cruzain inhibitor reveals a second target and a mammalian off-target
Source: Beilstein J Org Chem. 2013 Jan 4;9:15–25. doi: 10.3762/bjoc.9.3 (PMC3566858; doi:10.3762/bjoc.9.3)
Supplement: File 1 — Figures, schemes, and experimental procedures. [file Beilstein_J_Org_Chem-09-15-s001.pdf]

Supporting Information

for

Chemical–biological characterization of a cruzain inhibitor reveals a second target and a mammalian off-target

Jonathan W. Choy[1,4,5], Clifford Bryant[1,4], Claudia M. Calvet[2,3,6], Patricia S. Doyle[2,3], Shamila S. Gunatilleke[2,3], Siegfried S. F. Leung[4], Kenny K. H. Ang[1,4], Steven Chen[1,4], Jiri Gut[2,3], Juan A. Oses-Prieto[4], Jonathan B. Johnston[4], Michelle R. Arkin[1,2,4], Alma L. Burlingame[4], Jack Taunton[5], Matthew P. Jacobson[4], James M. McKerrow[2,3], Larissa M. Podust[2,3] and Adam R. Renslo[1,2,4]*

Address: [1]Small Molecule Discovery Center, [2]Center for Discovery and Innovation in Parasitic Diseases, [3]Department of Pathology, [4]Department of Pharmaceutical Chemistry, [5]Department of Cellular and Molecular Pharmacology, University of California San Francisco, 1700 4th Street, San Francisco, CA, 94158, USA and [6]Cellular Ultra-Structure Laboratory, Oswaldo Cruz Institute (IOC), FIOCRUZ, Rio de Janeiro, RJ, Brazil 21040-362

Email: Adam R. Renslo* - adam.renslo@ucsf.edu

*Corresponding author

Figures, schemes, and experimental procedures

Table of contents

Supplemental table S2

Supplemental figures S3–S4

Supplemental schemes S5–S6

Experimental procedures S7–S23

References S23–S24

Supplemental table

Table S1: Rank ordering of experimentally and computationally determined binding affinities for compounds **5**, **4**, **13**,and **12**.

Compound	K_D (nM)	Docking Score (Glide XP)	MM/GBSA score
**5**	≤5	−10.8	−84.2
**4**	≤5	−10.6	−91.9
**13**	75 ± 26	−11.5	−77.9
**12**	615 ± 264	−9.71	−63.9

Supplemental figures

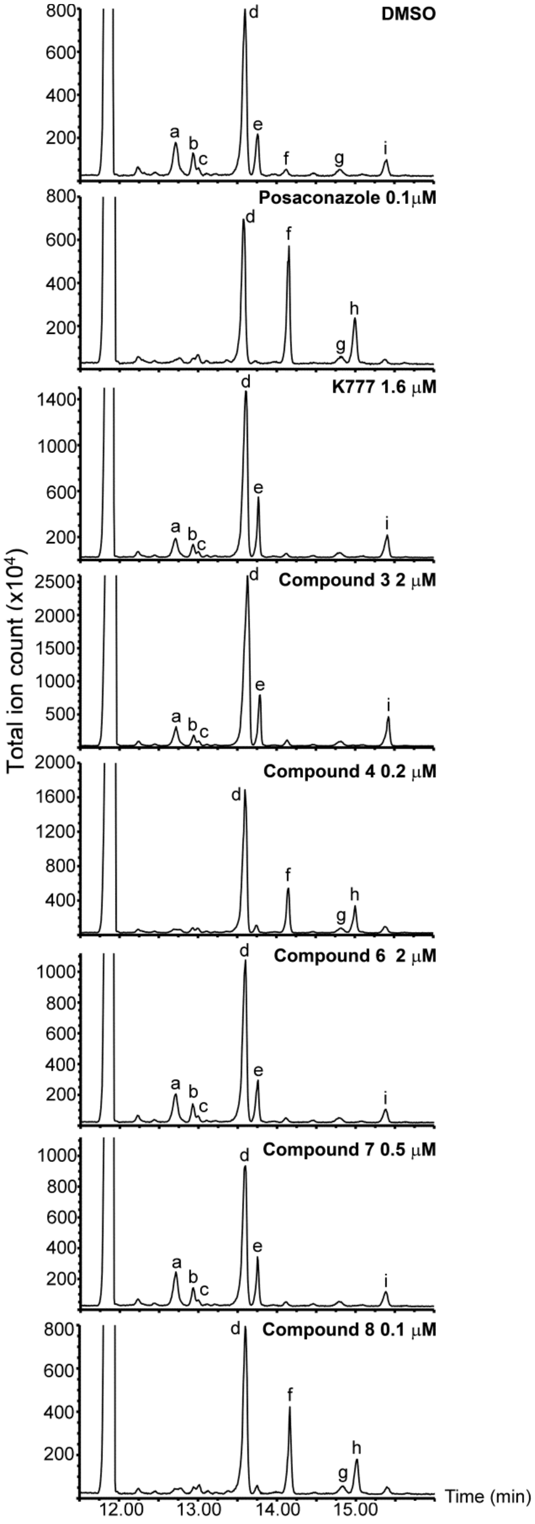

Figure S1: GC–MS spectra showing sterol composition of intra-cellular *T. cruzi* parasites treated with indicated test compounds. The analysis of **3–8** was performed concurrently with

other CYP51 inhibitors described recently [1] and, thus, the spectra for the controls shown above are reproduced from the earlier report. Peaks are assigned as **a** - cholesta-7,24-dien-3β-ol, $[M]^{\bullet+}$ = *m*/*z* 454; **b** - cholesta-8,24-dien-3β-ol (zymosterol), $[M]^{\bullet+}$ = *m*/*z* 470; **c** - 24-methyl-7-en-cholesta-en-3β-ol, $[M]^{\bullet+}$ = *m*/*z* 472; **d** - ergosta-7,24-diene-3β-ol (episterol), $[M]^{\bullet+}$ = *m*/*z* 470; **e** - ergosta-8,24-diene-3β-ol (fecosterol), $[M]^{\bullet+}$ = *m*/*z* 470; **f** - lanosterol, $[M]^{\bullet+}$ = m/z 498; **g** - 4-methylepisterol, $[M]^{\bullet+}$ = *m*/*z* 484; **h** - eburicol, $[M]^{\bullet+}$ = *m*/*z* 512; **i** - 24-ethyl-7,24(24')-en-cholesta-dien-3β-ol, $[M]^{\bullet+}$ = *m*/*z* 484.

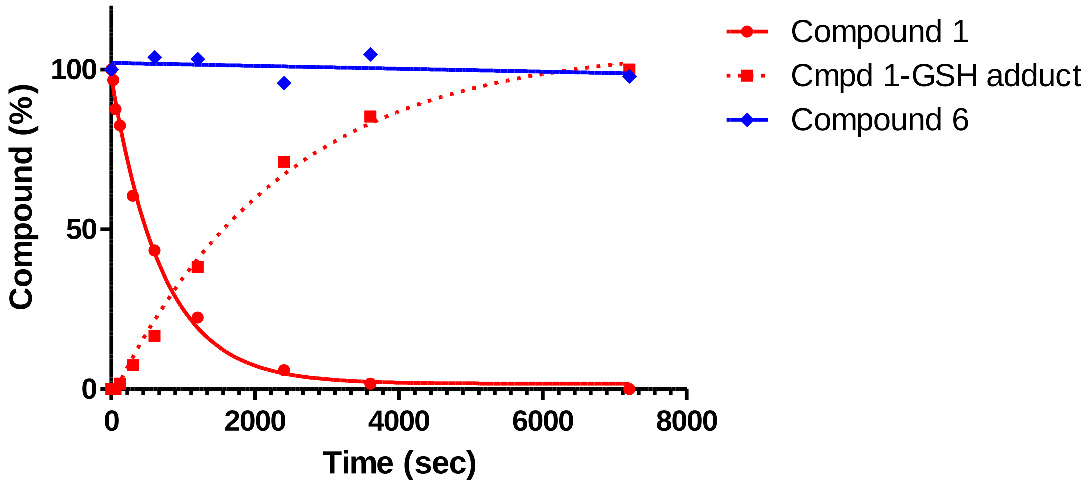

Figure S2: Time-course of reaction between compounds **1** or **6** with 10 mM glutathione in vitro.

Supplemental schemes

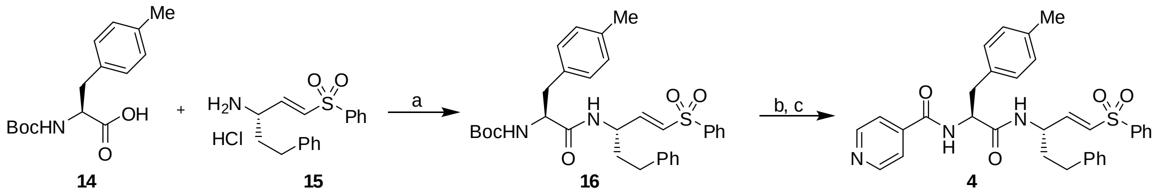

Scheme S1: Synthesis of compound **4**. Conditions: (a) HATU, DIEA, DMF; (b) HCl, dioxane; (c) Pyridyl acid, HATU, DIEA, DMF.

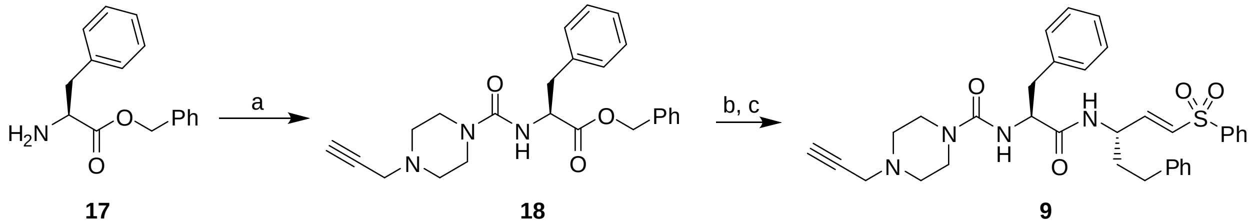

Scheme S2: Synthesis of activity-based probe **9**. Conditions: (a) triphosgene, CH_2Cl_2, aq. $NaHCO_3$, then *N*-propargylpiperazine; (b) NaOH, MeOH; (c) **15**, HATU, DMF, 11% overall.

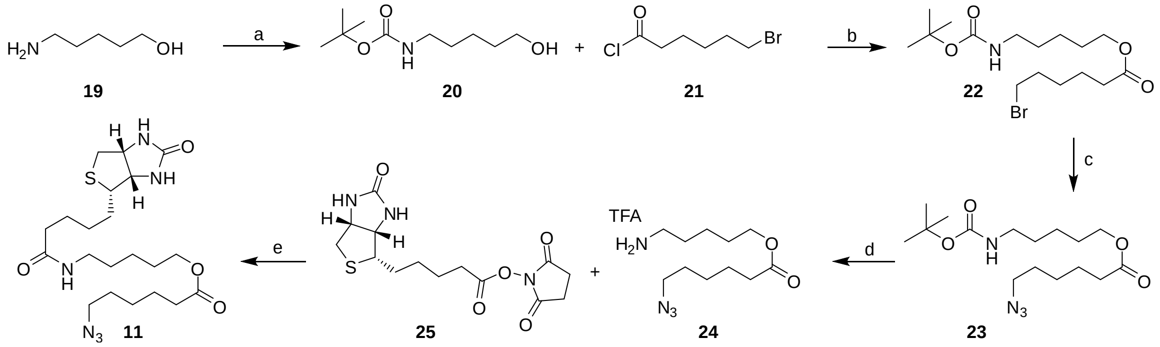

Scheme S3: Synthesis of cleavable biotin azide reagent **11**. Conditions: (a) di-*tert*-butyl dicarbonate, H_2O, NaOH, 88%; (b) CH_2Cl_2, Et_3N; (c) NaN_3, DMF, 49% over two steps; (d) TFA, CH_2Cl_2; (e) DMF, Et_3N, 59% over two steps.

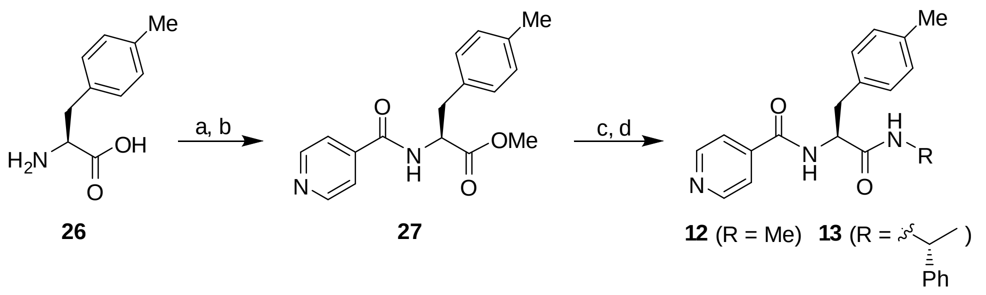

Scheme S4: Synthesis of analogues **12** and **13**. Conditions: (a) $SOCl_2$, MeOH; (b) isonicotinic acid, HATU, DIEA, DMF; (c) LiOH, THF, water; (d) R-NH_2, HATU, DIEA, DMF.

Experimental procedures

Computational modeling

The binding of compounds **4**, **5**, **12**, and **13** to CYP51 was modeled by using the induced fit docking software (Schrödinger Inc.). As previously described [2], these compounds were docked to a structural model that was developed based on a crystal structure of *Tc*CYP51 (PDB code 2WUZ) [3]. In brief, the protein and the compounds were first prepared for docking by using the Protein Preparation Wizard and the Ligprep modules, respectively. The Fe^{3+} ion of the heme group was defined as a metal site during the docking calculations. The induced fit docking protocol was employed to address protein flexibility through the following procedures [4]. Binding models were first generated by rigid docking with a softened potential using the GLIDE standard precision (SP) scoring function [5]. Models with top docking scores were then subjected to binding-site optimization in which amino acid residues within 5 Å from the docked compound were energetically minimized in the presence of the ligand by using PRIME [6]. The resulting low-energy complex structures that were within 30 kcal/mol from the lowest-energy complex were used in the subsequent redocking calculations. Each compound was redocked to these “induced fit” protein models and was evaluated by using the GLIDE extra precision (XP) scoring function. Finally, the predicted binding mode with the most favorable docking score was further refined by using the PRIME MM-GBSA protocol by energy minimizing the ligand and the surrounding protein residues within 5 Å [7].

TcCYP51 Binding assay

All K_D values were determined by using the UV–vis binding assay described previously [1]. Although CYP51 concentrations of 0.5 μM are required in this assay, application of the Morrison equation [8] allows us to estimate ligand binding with a lower limit of K_D ~5 nM.

CYP Inhibition and reversibility studies

Mammalian CYP inhibition values and reversibility studies were conducted by WuXi AppTech (Tianjin, China 300457) according to standard protocols.

Cruzain kinetics

Cruzain (0.1nM) in assay buffer (100mM sodium acetate pH 5.5, 5 mM DTT, 10 mM EDTA, and 0.01% Triton-X 100) was added to inhibitor dilutions in equal volume of 1 μM Z-Phe-Arg-AMC (Bachem) in the same buffer. Total assay volume is 20 μL in 384-well plate format or 200 μL in 96-well plate format. Progress curves were determined by using a Flexstation 3 fluorescent plate reader (Molecular Devices) with an excitation wavelength of 355 nm and an emission wavelength of 460 nm for 360 seconds at room temperature; for inhibitor concentrations ranging from 25–0.001 μM. Inhibitor dilutions that produced exponential progress curves over a wide range of k_{obs} were used to determine kinetic parameters. Using Prism5 software (GraphPad), the value of k_{obs}, the rate constant for loss of enzymatic activity, was determined from an equation for pseudo-first-order dynamics ($P = (\nu_i/k_{obs})[1 - \exp(-k_{obs}*t)]$); where product formation = P, initial rate = ν_i, time = t, and the first-order rate constant = k_{obs}). For inhibitors where k_{obs} varied hyperbolically with [I], nonlinear regression analysis was performed with Prism5 to determine k_{inact}/K_i using $k_{obs} = k_{inact}[I]/([I] + K_{i^*}(1 + [S]/K_m))$. IC_{50} values were determined by using a sigmoidal dose-response variable-slope model.

Minimum trypanocidal concentration assay

The minimal trypanocidal concentration assay was derived from the established protocol [9] for evaluating "cidal" activity, except that each compound was evaluated at multiple concentrations (typically 7 or 10) ranging from 0 to 50 μM. As in the original protocol, *T. cruzi* infected J744 macrophages were treated with test compound for 27 days (or until *T. cruzi* parasites appear).

During the course of treatment, media containing the test compound was replaced every 72 hours. After 27 days of treatment, the cultures are maintained under drug-free conditions until day 40. The MTC was taken as the lowest concentration of test compound that prevents reappearance of *T. cruzi* parasites as read at day 40.

Imaging-based screening assay

Antiparasitic EC_{90} values were determined by using an adaption [1] of the high-content screening approach described previously [10]. The assay employed mouse C2C12 myoblasts (ATCC#CRL-1772) infected with *T. cruzi* CAI-72 [11] trypomastigotes.

Ergosterol biosynthesis – GC–MS methods

To grow intracellular amastigotes for lipid analysis, C2C12 mouse skeletal myoblasts (4×10^6 cells) cultured in 150 cm^2 flasks as described elsewhere [1] were infected with 80×10^6 trypomastigotes for 24 hours. The cultures were treated with test compounds 72 hours post-infection at concentrations ~2–4-fold below their MTC, so as to retain a viable parasite population for analysis. Posaconazole at 100 nM was used as a positive control. The cysteine protease cruzain inhibitor K777 [12] at 1.6 μM was used as a negative control to ensure that *T. cruzi* inhibition via other pathways did not affect composition of the membrane sterols. Cells were harvested 96 hours post-infection and after 24 hours of drug treatment.

Gas chromatography/mass spectrometry (GC–MS) was used for lipid analysis as described previously [1]. Our GC–MS-based assays measure total sterol abundances, and allow us to delineate the effects of individual compounds on the ergosterol biosynthesis pathway of *T. cruzi*. Total lipids were extracted by treating the dry cell pellets with 2 mL chloroform for 24 hours. Polar lipids were removed by washing the organic phase thrice with 3 mL water. The organic phase was retained and subsequently dried on an evaporator under a stream of nitrogen gas.

The sterol-containing residue was resuspended in 2 mL of chloroform-methanol (9:1 ratio), washed thrice with 3 mL water, dried under N_2 gas and resuspended in acetonitrile. The acetonitrile phase was dried under a stream of N_2 and then washed with three portions of water (3 × 3 mL). The extracted sterols were dissolved in hexane (50 μL) and derivatized with 75 μL *N*,*N*-bis(trimethylsilyl)-2,2,2-trifluoroacetamide (BSTFA) (Pierce) by incubating at 37 °C for 2 h. Sterols were analyzed by GC–MS as the respective trimethylsilyl (TMS) derivatives using an Agilent HP 6850 GC system coupled with an Agilent 5979 mass-selective detector (MSD) operating at 70 eV. The individual sterols were separated using a DB-5-MS column (30 m × 0.25 mm inner diameter, 0.25-m film thickness) in which the temperature was held at 200 °C for 1 min, followed by an increase of 15 °C/min up to 300 °C and finally held at 300 °C for 20 min.

Cellular labeling assay and "click" chemistry: C2C12 cells (1.5 × 10^6 cells) were pre-treated with competitor compounds in culture media (1 mL, DMEM with 10% FBS) for 1 hour at 37 °C. Then additional competitor compound and **9** (2 μM) were added in culture media (1 mL), and the cells were incubated for an additional hour. The cells were then pelleted, washed with phosphate-buffered saline pH 7.4 (2 × 1 mL), and lysed with 1% SDS in phosphate-buffered saline (400 μL). The denatured lysates were sonicated to reduce viscosity, centrifuged to give pellets of the insoluble material, and normalized for total protein content. The normalized lysates (22 μL, 3 mg/mL) were mixed with TAMRA azide **10** (0.5 μL, 5 mM), TCEP (0.5 μL, 50 mM, pH ~7.0), TBTA ligand in 1:4 DMSO/*t*-butyl alcohol (1.5 μL, 1 mM), and $CuSO_4$ (0.5 μL, 50 mM). Reactions were incubated at room temperature for one hour and then resolved by SDS-PAGE. The resulting gel was scanned for fluorescence (Typhoon Imaging System, Molecular Dynamics) and coomassie stained.

Enrichment of 9-labeled proteins and mass spectrometric analysis. C2C12 cells were treated with probe **9**, lysed and denatured as described above. The labeled and denatured

lysates were sonicated to reduce viscosity and centrifuged to give pellets of the insoluble material. The buffer was then exchanged by using a NAP-5 column (GE Healthcare) equilibrated with 1% SDS in phosphate buffered saline pH 7.4. The samples were then normalized for protein content. The normalized lysates (1 mL, 2 mg/mL) were subjected to a "click" reaction with a cleaveable biotin azide **11** using the general conditions described above. Samples were enriched using Streptavidin-coupled Dynabeads (Invitrogen). Enriched proteins were eluted with aqueous sodium hydroxide (0.4 N). The solution was neutralized with aqueous hydrochloric acid (0.8 N), and then run on an SDS-PAGE gel. The gel was colloidal coomassie stained.

The competed band at ~35 kDa was cut, and the proteins contained in the bands of interest were digested in gel with trypsin as described previously [13]. Using the protocol we described recently [14], the extracted digests were vacuum-evaporated and resuspended in 10 µL of 0.1% formic acid in water. The digests were separated by nanoflow liquid chromatography using a 75 µm × 150 mm reverse-phase 1.7 µm BEH 130 C18 column (Waters) at a flow rate of 350 nL/min in a NanoAcquity^{TM} Ultra performance UPLC system (Waters). Solvent A was 0.1% formic acid in water and solvent B was 0.1% formic acid in acetonitrile. Following equilibration of the column in 5% solvent B, approximately one-half of each digest (5 µL) was injected, then the organic content of the mobile phase was increased linearly to 40% over 60 min, and then to 50% in 1 min. The liquid chromatography eluate was coupled to a hybrid linear ion trap-Orbitrap mass spectrometer (LTQ-XL, Thermo Scientific, San Jose, CA) equipped with a nanoelectrospray ion source. Spraying was from an uncoated 15 µm inner diameter spraying needle (New Objective, Woburn, MA). Peptides were analyzed in positive ion mode and in information-dependent acquisition mode to automatically switch between MS and MS/MS acquisition. MS spectra were acquired in profile mode using the Orbitrap analyzer in the *m*/*z* range between 300 and 1800. For each MS spectrum, the 6 most intense multiple charged ions over a threshold of 1000

counts were selected to perform CID (collision-induced dissociation) experiments. Product ions were analyzed on the linear ion trap in centroid mode. The CID collision energy was automatically set to 25%. A dynamic exclusion window of 0.5 Da was applied that prevented the same *m*/*z* from being selected for 60 seconds after its acquisition.

Mass spectrometry data analysis. Peak lists were generated using PAVA [15]. The peak lists were searched against the murine and human subset of the UniProtKB database as of July 6, 2011 (containing 182779 entries) using ProteinProspector version 5.8.0. Peptide tolerance in searches was 20 ppm for precursor and 0.6 Da for product ions, respectively. Cleavage specificity was selected to Trypsin. Peptides containing two miscleavages were allowed. Carbamidomethylation of cysteine, acetylation of the N-terminus of the protein, pyroglutamate formation from N-terminal glutamine, and oxidation of methionine were allowed as variable modifications.

The number of modifications was limited to two per peptide. Hits were considered significant when two or more peptide sequences matched a protein entry and the Prospector score was above the significance level. A minimal ProteinProspector protein score of 20, a peptide score of 15, a maximum expectation value of 0.05 and a minimal discriminate score threshold of 0.0 were used for initial identification criteria. For identifications based on one peptide sequence with high scores, the MS/MS spectrum was reinterpreted manually by matching all the observed fragment ions to a theoretical fragmentation obtained by using MS Product (Protein Prospector).

Reactivity with glutathione

Compounds **1** and **6** were incubated with reduced glutathione (10mM) in phosphate-buffered saline (pH 7.4). The reactions were quenched with a solution of trifluoroacetic acid (2%) in methanol and then separated by reverse-phase liquid chromatography. The amount of

unreacted compound remaining was quantified by integrating the compound peak observed by evaporative light scattering. This value was normalized to an internal control of quinine, and then used to report the fraction of compound remaining.

Synthetic procedures.

^{1}H NMR spectra were recorded on a Varian INOVA-400 400 MHz spectrometer. Chemical shifts are reported in δ units (ppm) relative to residual solvent peaks. Coupling constants (*J*) are reported in hertz (Hz). Compounds **1**, **2**, and **3** were prepared as described previously [16] and TAMRA azide **10** was prepared according to the reported procedure [17]. Compound **9** was prepared as described below and exhibited spectral characteristics consistent with those reported previously [18]. Intermediate **15** was prepared as described previously [19]. All reagents and solvents were purchased from Aldrich Chemical or Acros Organics and used as received. Column chromatography was carried out by using a Biotage SP1 flash-chromatography system and silica-gel cartridges from Biotage. Analytical TLC plates from EM Science (Silica Gel 60 F254) were employed for TLC analyses. Preparative HPLC purifications were performed by using a Biotage Parallex Flex equipped with Waters Xbridge 19 × 50mm C18 5μM OBD columns.

***tert*-Butyl *N*-[(2*S*)-1-[[(*E*,3*S*)-1-(benzenesulfonyl)-5-phenylpent-1-en-3-yl]amino]-3-(4-methylphenyl)-1-oxopropan-2-yl]carbamate (16).**

(2S)-3-(4-Methylphenyl)-2-[(2-methylpropan-2-yl)oxycarbonylamino]propanoic acid (**14**, 0.040 g, 0.143 mmol) and (1*E*,3*S*)-1-(benzenesulfonyl)-5-phenylpent-1-en-3-amine hydrochloride (**15**, 0.051 g, 0.150 mmol) were dissolved in 1 mL DMF. HATU (0.086 g, 0.225 mmol) was added, followed by diisopropylethylamine (0.150 mL, 0.858 mmol). The reaction mixture was stirred at room temperature until complete as judged by LC-MS. The reaction mixture was diluted with 5

mL ethyl acetate, shaken with 5 mL 1N HCl, 5 mL 50% saturated $NaHCO_3$ then 5 mL brine. The organic layer was further dried with $MgSO_4$. Solvent was removed to afford the crude product (89 mg), which was used without further purification. ^{1}H NMR (400 MHz, $CDCl_3$) δ 1.26–1.54 (m, 9 H), 1.74–1.96 (m, 2 H), 2.28 (s, 3 H), 2.49–2.64 (m, 2 H), 2.91–3.01 (m, 2 H), 4.18- 4.28 (m, 1 H), 4.59–4.71 (m, 1 H), 4.97 (br s, 1 H), 6.11–6.22 (m, 2 H), 6.79 (dd, *J* = 15.11, 4.67 Hz, 1 H), 6.98–7.11 (m, 5 H), 7.14–7.20 (m, 1 H), 7.19–7.25 (m, 1 H), 7.48–7.54 (m, 2 H), 7.57–7.66 (m, 1 H), 7.81–7.85 (m, 1 H).

General procedure for preparing pyridyl analogs 3 and 4.

tert-Butyl *N*-[(2*S*)-1-[[(*E*,3*S*)-1-(benzenesulfonyl)-5-phenylpent-1-en-3-yl]amino]-3-(4-methylphenyl)-1-oxopropan-2-yl]carbamate (**16**) is treated with 4N HCl in dioxane (~100 molar equiv HCl) at room temperature until cleavage if the Boc group is complete, as judged by LC/MS analysis. The solvent is then removed and the residue resuspended in DMF (~0.15 M). The requisite pyridine carboxylic acid (1.2 equiv), HATU (1.5 equiv) and diisopropylethylamine (6.0 equiv) are added and the reaction mixture stirred until the coupling is judged complete by LC/MS analysis, typically 12 hours. The reaction mixture is then diluted with ethyl acetate (at least 5x volume) and shaken with an equivalent volume of 50% saturated aqueous $NaHCO_3$. The organic layer is separated and then washed with saturated NaCl, and dried ($MgSO_4$), filtered, and concentrated to afford the crude product, which is purified using automated silica gel chromatography with a mobile phase of ethyl acetate/hexanes.

***N*-[(2*S*)-1-[[(*E*,3*S*)-1-(Benzenesulfonyl)-5-phenylpent-1-en-3-yl]amino]-3-(4-methylphenyl)-1-oxopropan-2-yl]pyridine-2-carboxamide trifluoroacetate salt (3).**

Compound **3** was prepared according to the general procedure except that additional purification by HPLC was required. 10% overall yield from **14** ^{1}H NMR (400 MHz, $CDCl_3$) δ

1.71–1.82 (m, 1 H), 1.82–1.92 (m, 1 H), 2.29 (s, 3 H), 2.48–2.57 (m, 2 H), 3.16 (dd, *J* = 7.05, 2.11 Hz, 2 H), 4.65 (dd, *J* = 5.13, 3.48 Hz, 1 H), 4.75 (q, *J* = 7.33 Hz, 1 H), 6.22 (dd, *J* = 15.11, 1.74 Hz, 1 H), 6.39 (d, *J* = 7.87 Hz, 1 H), 6.80 (dd, *J* = 15.11, 4.85 Hz, 1 H), 6.96–7.02 (m, 2 H), 7.02–7.09 (m, 2 H), 7.09–7.22 (m, 4 H), 7.48 (ddd, *J* = 7.55, 4.81, 1.01 Hz, 1 H), 7.52–7.60 (m, 2 H), 7.60–7.67 (m, 1H), 7.82–7.92 (m, 2 H), 8.14 (d, *J* = 7.87 Hz, 1 H), 8.53–8.66 (m, 2 H); ^{13}C NMR (100 MHz, $CDCl_3$) δ 21.1, 31.7, 35.5, 37.3, 49.3, 55.3, 122.7, 126.2, 126.8, 127.6, 128.3, 128.5, 129.1, 129.3, 129.5, 130.6, 133.2, 133.4, 136.9, 138.0, 140.2, 140.3, 145.5, 147.9, 148.6, 164.3, 170.4; MS: (*m/z*): $[M + H]^+$ = 568.6

***N*-[(2*S*)-1-[[(*E*,3*S*)-1-(Benzenesulfonyl)-5-phenylpent-1-en-3-yl]amino]-3-(4-methylphenyl)-1-oxopropan-2-yl]pyridine-4-carboxamide (4).**

Compound **4** was prepared according to the general procedure in 45% overall yield from **14**. ^1H NMR (400 MHz, $CDCl_3$) δ1.71–1.84 (m, 1 H), 1.84–1.97 (m, 1 H), 2.23 (s, 1 H), 2.34 (s, 2 H), 2.46–2.64 (m, 2 H), 2.77–2.86 (m, 1 H), 3.01 (dd, *J* = 13.46, 8.88 Hz, 1 H), 3.17 (dd, *J* = 13.92, 5.86 Hz, 1 H), 4.59–4.73 (m, 1 H), 5.75 (d, *J* = 8.24 Hz, 1 H), 6.19 (d, *J* = 1.65 Hz, 1 H), 6.15 (d, *J* = 1.47 Hz, 1 H), 6.77 (d, *J* = 5.49 Hz, 1 H), 6.73 (d, *J* = 5.31 Hz, 1 H), 6.87 (d, *J* = 7.33 Hz, 1 H), 6.99–7.29 (m, 7 H), 7.43–7.72 (m, 3 H), 7.72–8.00 (m, 2 H), 8.62–8.84 (m, 2 H); MS: (*m/z*): $[M + H]^+$ = 568.6

General procedure for reduction of 1, 3 and 4 to afford analogs 6, 7 and 8.

The vinylsulfone starting material was dissolved in ethanol (0.015 mM concentration) and one weight equivalent of 10% Pd on C was added. The flask was evacuated and filled with hydrogen three times, and the reaction mixture was maintained under a hydrogen atmosphere (1 atm) until the vinyl group was fully reduced, but before over-reduction of the pyridyl ring began to occur. Typically this required ~6 h. The hydrogen balloon was then removed and the reaction

mixture filtered through Celite. The filtrate was concentrated and the crude product purified by using automated silica-gel chromatography with a mobile phase of ethyl acetate/hexanes.

***N*-[(2*S*)-1-[[(3*S*)-1-(Benzenesulfonyl)-5-phenylpentan-3-yl]amino]-1-oxo-3-phenylpropan-2-yl]-4-methylpiperazine-1-carboxamide (6).**

Compound **6** was prepared from **1** according to the general procedure, except that additional purification by preparative HPLC was required. ^{1}H NMR (400 MHz, $CDCl_3$) δ 1.53–1.83 (m, 4 H), 2.35–2.61 (m, 2 H), 2.61–2.85 (m, 3 H), 2.86–3.03 (m, 3 H), 3.09 (dd, *J* = 13.73, 6.96 Hz, 1 H), 3.28 (br s, 2 H), 3.45 (br s, 1 H), 3.75–3.91 (m, 1 H), 3.96 (br s, 1 H), 4.05 (br s, 2 H), 4.29–4.46 (m, 1 H), 6.27 (d, *J* = 6.59 Hz, 1 H), 6.90 (d, *J* = 8.97 Hz, 1 H), 7.04 (d, *J* = 6.96 Hz, 2 H), 7.08–7.35 (m, 7 H), 7.53 (t, *J* = 7.78 Hz, 2 H), 7.64 (t, *J* = 7.42 Hz, 1 H), 7.72–7.91(m, 2 H); MS: (*m/z*): $[M + H]^+$ = 577.2

***N*-[(2*S*)-1-[[(3*S*)-1-(Benzenesulfonyl)-5-phenylpentan-3-yl]amino]-3-(4-methylphenyl)-1-oxopropan-2-yl]pyridine-2-carboxamide (7).**

Compound **7** was prepared from **3** according to the general procedure. Yield: 20% ^{1}H NMR (400 MHz, $CDCl_3$) δ 1.55–1.73 (m, 3 H), 1.82–1.95 (m, 1 H), 2.19–2.35 (m, 3 H), 2.37–2.51 (m, 2 H), 2.84–3.04 (m, 2H), 3.04–3.24 (m, 2 H), 3.88 (tq, *J* = 9.21, 4.36 Hz, 1 H), 4.59–4.74 (m, 1 H), 5.96 (d, *J* = 9.16 Hz, 1 H), 6.93–7.00 (m, 2 H), 7.03–7.22 (m, 6 H), 7.44 (ddd, *J* = 7.55, 4.81, 1.19 Hz, 1 H), 7.51–7.59 (m, 2 H), 7.60–7.68 (m, 1 H), 7.80–7.89 (m, 2 H), 8.13 (dt, *J* = 7.83, 1.03 Hz, 1 H), 8.47–8.59 (m, 2 H); MS: (*m/z*): $[M + H]^+$ = 570.2

***N*-[(2*S*)-1-[[(3*S*)-1-(Benzenesulfonyl)-5-phenylpentan-3-yl]amino]-3-(4-methylphenyl)-1-oxopropan-2-yl]pyridine-4-carboxamide (8).**

Compound **8** was prepared from **4** according to the general procedure. Yield: 26% ^{1}H NMR (400 MHz, $CDCl_3$) δ 8.72 (br s, 2H), 7.84–7.90 (m, 2H), 7.65–7.71 (m, 1H), 7.57–7.61 (m, 2H), 7.52–7.57 (m, 2H), 7.16–7.23 (m, 3H), 7.11–7.16 (m, 4H), 6.99–7.03 (m, 2H), 6.96 (d, *J* = 7.33 Hz, 1H), 5.82 (d, *J* = 8.97 Hz, 1H), 4.70 (dt, *J* = 6.23, 7.87 Hz, 1H), 3.90 (td, *J* = 4.56, 9.02 Hz, 1H), 3.04–3.19 (m, 2H), 2.85–3.02 (m, 2H), 2.42–2.55 (m, 2H), 2.34 (s, 3H), 1.78–2.00 (m, 1H), 1.54–1.76 (m, 3H); ^{13}C NMR (100 MHz, $CDCl_3$) δ 170.4, 165.4, 150.6, 140.7, 140.6, 139.0, 137.3, 133.8, 132.9, 129.8, 129.3, 129.0, 128.5, 128.2, 128.0, 126.2, 120.8, 55.4, 52.9, 48.3, 37.8, 36.6, 32.0, 28.0, 21.1 MS: (*m/z*): $[M + H]^+$ = 570.2

Benzyl (2*S*)-3-phenyl-2-[(4-(prop-2-ynyl)piperazine-1-carbonyl)amino]propanoate (18).

Benzyl (2*S*)-2-amino-3-phenylpropanoate hydrochloride (**17**, 2.0 g, 6.85 mmol, 1.0 equiv) was dissolved in methylene chloride (70 mL), and the solution was cooled in an ice/water bath. A saturated aqueous solution of sodium bicarbonate (70 mL) was then added, followed by a solution of triphosgene (0.81 g, 2.74 mmol, 0.4 equiv) in methylene chloride (15 mL), which was added slowly. The reaction was stirred for 1 hour and then quenched by the addition of excess benzylamine. The aqueous layer was extracted with methylene chloride (2 × 50 mL) and the combined organic layers were then washed with 1M hydrochloric acid (2 × 10 mL). Hexane was added to precipitate the desired isocyanate intermediate (1.7 g, 89% crude yield) which contained a small amount of starting material. A portion of this material was used without further purification in the next reaction.

A flask was charged with 4-(prop-2-ynyl)piperazine dihydrochloride (120 mg, 0.61 mmol, 1.0 equiv) and dissolved in a mixture of tetrahydrofuran (1 mL), dimethylformamide (0.4 mL), and diisopropylethylamine (0.42 mL, 2.44 mmol, 4.0 equiv). In another flask, the crude benzyl (2*S*)-

2-isocyanato-3-phenylpropanoate (171 mg, 0.061 mmol, 1.0 equiv) was dissolved in tetrahydrofuran (1 mL) and chilled in an ice/water bath. The 4-(prop-2-ynyl)piperazine solution was then added dropwise to the solution of isocyanate. After 1 hour, the reaction was diluted with ethyl acetate (10 mL) and washed with water (5 × 5 mL) and brine (5 mL). The organic layer was then dried over sodium sulfate, filtered, and concentrated. The crude residue was subjected to automated flash chromatography using a solvent gradient of methanol in dichloromethane (0–4%). The relevant fractions were collected and concentrated to afford benzyl (2*S*)-3-phenyl-2-[(4-(prop-2-ynyl)piperazine-1-carbonyl)amino]propanoate (**18**, 195 mg, 79% yield, ~85% pure) as a clear gum. This material was used in the next step without further purification. MS: 406.15 [M + H].

***N*-[(2*S*)-1-[[(*E*,3*S*)-1-(Benzenesulfonyl)-5-phenylpent-1-en-3-yl]amino]-1-oxo-3-phenylpropan-2-yl]-4-prop-2-ynylpiperazine-1-carboxamide (9).**

A flask was charged with benzyl (2*S*)-3-phenyl-2-[(4-(prop-2-ynyl)piperazine-1-carbonyl)amino]propanoate (**18**, 90 mg, 0.22 mmol, 1.0 equiv), and methanol (2 mL) was added. Aqueous sodium hydroxide (2M, 0.24 mL) was added, and the resulting mixture was stirred overnight and then concentrated to dryness. The resulting crude (2*S*)-3-phenyl-2-[(4-(prop-2-ynyl)piperazine-1-carbonyl)amino]propanoic acid (MS: 316.04 (M+H)) was dissolved in dimethylformamide (1 mL) and treated with (*E*,3*S*)-1-(benzenesulfonyl)-5-phenylpent-1-en-3-amine (75 mg, 0.22 mmol, 1.0 equiv) and diisopropylethylamine (118 µL, 0.67 mmol, 3.0 equiv). The mixture was then cooled in a dry ice–acetone bath, and HATU (92.8 mg, 0.24 mmol, 1.1 equiv) was added in one portion. The reaction was allowed to slowly warm to −40 °C over 3 hours and was then diluted in ethyl acetate (20 mL) and washed with water (5 × 5 mL) and brine (5 mL). The organic layer was dried over sodium sulfate, filtered and concentrated. The crude material was then purified by reverse-phase HPLC, affording 17 mg of compound **2** as a white

solid (11% over three steps). ^{1}H NMR (400 MHz, $CDCl_3$) δ 1.79 (m, 1H), 1.89 (m, 1H), 2.50 (t, *J* = 2.1 Hz, 1H), 2.52–2.62 (m, 2H), 2.92–3.07 (m, 6H), 3.60 (m, 4H), 3.72 (m, 2H), 4.37 (q, *J* = 7.7Hz, 1H), 4.61 (m, 1H), 5.12 (m, 1H), 5.98 (m, 1H), 6.07 (dd, *J* = 15.4, 1.2 Hz, 1H), 6.75 (dd, *J* = 15.4, 5.3 Hz, 1H), 7.00–7.90 (m, 15H). LCMS: 599.17 (M+H); MW: 598.75).

***tert*-Butyl 5-hydroxypentylcarbamate (20)**: 5-Amino-1-pentanol (**19**, 500 mg, 4.85 mmol) and di-*tert*-butyl dicarbonate (881 mg, 4.04 mmol) were dissolved in water (5 mL). The solution was adjusted to pH 12 with 1N NaOH (aq) and stirred for 20 min. The reaction was extracted three times with ethyl acetate. The organic layers were combined, washed with brine, dried over sodium sulfate, and concentrated to afford the product as a clear oil (719 mg, 88% yield). ^{1}H NMR (400 MHz, $CDCl_3$) δ 1.30–1.70 (m, 15H), 3.12 (q, *J* = 6.5 Hz, 2H), 3.63 (m, 2H), 4.53 (b s, 1H).

5-(*tert*-Butoxycarbonylamino)pentyl 6-bromohexanoate (22): *tert*-Butyl 5-hydroxypentylcarbamate (**20**, 696 mg, 3.42 mmol) and triethylamine (416 mg, 4.11 mmol) were mixed in methylene chloride (5 mL) and cooled in an ice-water bath. The 6-bromohexanoyl chloride (**21**, 1.464 g, 6.06 mmol) in methylene chloride (5 mL) was added slowly to the reaction. After 20 min, water (5 mL) was added, and the aqueous layer was acidified with 1N HCl (aq). The organic layer was separated, washed with saturated $NaHCO_3$ (aq) and brine, dried over sodium sulfate, and concentrated under reduced pressure. A clear oil containing a mixture (2:1) of the desired product and the starting acid chloride was obtained after silica flash chromatography using a gradient of ethyl acetate up to 25% (v/v) in hexanes. The crude material was used without further purification. ^{1}H NMR (400 MHz, $CDCl_3$) δ 1.30–1.55 (m, 15H), 1.59–1.69 (m, 4H), 1.82–1.92 (m, 2H), 2.31 (t, *J* = 7.4 Hz, 2H), 3.11(q, *J* = 6.6 Hz, 2H), 3.40 (t, *J* = 6.8 Hz, 2H), 4.05 (t, *J* = 6.7 Hz, 2H), 4.51 (br s, 1H).

5-(*tert*-Butoxycarbonylamino)pentyl 6-azidohexanoate (23): Crude 5-(*tert*-butoxycarbonylamino)pentyl 6-bromohexanoate (**22**, 220mg) and sodium azide (301 mg, 4.63 mmol) were dissolved in dimethylformamide (10 mL). The vented mixture was stirred at 50 °C behind a blast shield for 48 h. The reaction was diluted in ethyl acetate (80 mL) and washed extensively with water. The organic layer was washed with brine, dried over sodium sulfate, and concentrated. Silica flash chromatography using a gradient of ethyl acetate up to 25% (v/v) in hexanes yielded the desired product as a yellow oil, (110 mg, 49% yield over 2 steps). ^{1}H NMR (400 MHz, $CDCl_3$) δ 1.30–1.55 (m, 15H), 1.55–1.70 (m, 6H), 2.30 (t, J = 7.4 Hz, 2H), 3.10 (q, J = 6.5 Hz, 2H), 3.26 (t, J = 6.9 Hz, 2H), 4.05 (t, J = 6.7 Hz, 2H), 4.50 (br.s, 1H).

5-Aminopentyl 6-azidohexanoate (24): 5-(*tert*-Butoxycarbonylamino)pentyl 6-azidohexanoate (**23**, 50 mg, 0.146 mmol) was dissolved in methylene chloride (0.6 mL) and cooled in an ice bath. Trifluoroacetic acid (0.4 mL) was added dropwise and then stirred for 20 min. Volatiles were removed under reduced pressure to afford the trifluoroacetic acid salt in quantitative yield as a yellow oil. ^{1}H NMR (400 MHz, $CDCl_3$): 1.34–1.47 (m, 4H), 1.55–1.75 (m, 8H), 2.31 (t, J = 7.5 Hz, 2H), 2.96 (m, 2H), 3.26 (t, J = 6.9 Hz, 2H), 4.05 (t, J = 6.6 Hz, 2H), 7.80(m, 3H).

Base cleavable biotin azide (11): 5-aminopentyl 6-azidohexanoate (**24**, TFA salt, 31.3 mg, 0.088 mmol), biotin-NHS ester (**25**, 20 mg, 0.059 mmol), triethylamine (17.8 mg, 0.175 mmol) were dissolved in dimethylformamide (0.8 mL) and stirred at room temperature for 48 h. The reaction was diluted into ethyl acetate (20 mL), washed three times with 0.5N HCl (aq), once with brine, dried over magnesium sulfate, and concentrated. Silica flash chromatography using stepwise 0.5% increments methanol to 10% (v/v) in methylene chloride yielded the desired product as a white solid (16.2 mg, 59% yield). ^{1}H NMR (400 MHz, $CDCl_3$): 1.29–1.79 (m, 16H), 2.18 (t, J = 7.4 Hz, 2H), 2.30 (t, J = 7.4 Hz, 2H), 2.72 (d, J = 12.8 Hz, 1H), 2.89 (dd, J = 4.8, 12.7

Hz, 1H), 3.13 (m, 2H), 3.21 (q, *J* = 6.5 Hz, 2H), 3.26 (t, *J* = 7.4 Hz, 2H), 4.05 (t, *J* = 6.7 Hz, 2H), 4.30 (dd, *J* = 7.4, 4.9 Hz, 1H), 4.50 (dd, *J* = 7.4, 5.0 Hz, 1H), 5.71 (br s, 1H), 6.09 (m, 1H), 6.39 (s, 1H). ^{13}C NMR (100 MHz, $CDCl_3$): 23.28, 24.49, 25.64, 26.22, 28.07, 28.23, 28.31, 28.53, 29.21, 34.06, 36.00, 39.28, 40.47, 51.21, 55.60, 60.29, 61.89, 64.14, 173.14, 173.57.

Methyl (2*S*)-3-(4-methylphenyl)-2-(pyridine-4-carbonylamino)propanoate (27).

To a flask containing anhydrous methanol (5.0 mL) and cooled in an ice bath was added thionyl chloride (0.71 mL, 9.77 mmol, 3.5 equiv). After stirring for 5 minutes, (2*S*)-2-amino-3-(4-methylphenyl)propanoic acid (**26**, 0.50 g, 2.79 mmol) was added and the reaction mixture was warmed to room temperature with stirring for 64 hours. The solvent was evaporated and the residue was triturated twice with ether to afford methyl (2*S*)-2-amino-3-(4-methylphenyl)propanoate hydrochloride (0.61 g, 95%).

^{1}H NMR (400 MHz, D_2O) δ 2.31 (s, 3 H), 3.19 (dd, *J* = 14.47, 7.33 Hz, 1 H), 3.28 (dd, *J* = 14.56, 5.95 Hz, 1 H), 3.82 (s, 3 H), 4.39 (dd, *J* = 7.33, 6.04 Hz, 1 H), 7.11–7.21 (m, 2 H), 7.21–7.30 (m, 2 H).

A solution of (2*S*)-2-amino-3-(4-methylphenyl)propanoate hydrochloride (0.61 g, 2.67 mmol) in DMF (10 mL) was treated with isonicotinic acid (0.39 g, 3.20 mmol), HATU (1.52 g, 4.01 mmol) and diisopropylethylamine (2.32 mL, 13.4 mmol) and the reaction mixture stirred overnight. The reaction mixture was then poured into ethyl acetate and washed with 50% saturated $NaHCO_3$, saturated NaCl, and dried ($MgSO_4$), filtered, and concentrated. The crude product was purified by automated silica-gel chromatography to afford **27** (0.36 g, 45% overall) ^{1}H NMR (400 MHz, $CDCl_3$) δ 2.24–2.39 (m, 3 H), 3.21 (qd, *J* = 13.83, 5.40 Hz, 2 H), 3.73–3.83 (m, 3 H), 4.96–5.12 (m, 1 H), 6.61 (d, *J* = 6.41 Hz, 1 H), 6.98 (d, *J* = 7.69 Hz, 2 H), 7.09 (d, *J* = 7.87 Hz, 2 H), 7.49–7.63 (m, 2 H), 8.65–8.80 (m, 2 H).

General procedure for the synthesis of 12 and 13 from 27.

Intermediate **27** (0.36 g, 1.22 mmol) was dissolved in 5 mL 4:1 THF/water and then lithium hydroxide (0.117 g, 4.86 mmol) was added. The reaction mixture was stirred at room temperature and usually forms a suspension. When the hydrolysis reaction was judged to be complete by LC/MS, the reaction mixturewas adjusted to pH ~4 by slow addition of ~0.8 mL 2N HCl. The solvent was then removed in vacuo, and the crude lithium carboxylate intermediate was used in subsequent coupling reactions without further purification.

A portion of the lithium carboxylate intermediate was dissolved in DMF (0.15 M) and the requisite amine (2.2 equiv), HATU (2.0 equiv), and diisopropylethylamie (5.0 equiv) were added. The reaction mixture was stirred at room temperature until the reaction was judged complete by LC/MS (typically 2 h). The reaction mixture was then diluted with ethyl acetate (at least 5x volume) and shaken with an equivalent volume of 50% saturated $NaHCO_3$. The organic layer was then separated, washed with saturated NaCl, dried ($MgSO_4$), filtered, and concentrated. The crude product was then purified by automated silica-gel chromatography (ethyl acetate/hexanes) and/or preparative HPLC to afford the final product.

***N*-[(2*S*)-1-(Methylamino)-3-(4-methylphenyl)-1-oxopropan-2-yl]pyridine-4-carboxamide (12).**

Prepared according to the general procedure, employing methylamine in the coupling reaction. Yield: 33% ^{1}H NMR (400 MHz, $CDCl_3$) δ 2.15–2.33 (m, 3 H), 2.60–2.77 (m, 3 H), 2.97–3.12 (m, 1 H), 3.12–3.24 (m, 1 H), 4.78 (td, J = 7.83, 6.50 Hz, 1 H), 6.15 (d, J = 4.58 Hz, 1 H), 6.93–7.17 (m, 3 H), 7.46–7.63 (m, 3 H), 8.53–8.72 (m, 2 H); MS: (*m/z*): $[M + H]^+$ = 298.1

***N*-[(2*S*)-3-(4-Methylphenyl)-1-oxo-1-[[(1*R*)-1-phenylethyl]amino]propan-2-yl]pyridine-4-carboxamide (13).**

Prepared according to the general procedure, employing (*R*)-phenethylamine in the coupling reaction. Yield: 43% ^{1}H NMR (400 MHz, $CDCl_3$) δ 1.29 (d, *J* = 6.96 Hz, 2 H), 2.36 (s, 2 H), 3.08 (dd, *J* = 13.37, 8.97 Hz, 1 H), 3.27 (dd, *J* = 13.37, 6.04 Hz,1 H), 4.69–4.87 (m, 1 H), 4.95 (quin, *J* = 6.96 Hz, 1 H), 6.15 (d, *J* = 7.33 Hz, 2 H), 6.25 (br. s., 2 H), 7.08–7.38 (m, 7 H), 7.85 (br s, 2 H), 8.15 (d, *J* = 7.14 Hz, 1 H), 8.63 (br s, 2 H); MS: (*m/z*): $[M + H]^+$ = 388.2

References

1. Gunatilleke, S. S.; Calvet, C. M.; Johnston, J. B.; Chen, C. K.; Erenburg, G.; Gut, J.; Engel, J. C.; Ang, K. K.; Mulvaney, J.; Chen, S.; Arkin, M. R.; McKerrow, J. H.; Podust, L. M. *PLoS Negl. Trop. Dis.* **2012**, *6*, e1736.
2. Doyle, P. S.; Chen, C. K.; Johnston, J. B.; Hopkins, S. D.; Leung, S. S.; Jacobson, M. P.; Engel, J. C.; McKerrow, J. H.; Podust, L. M. *Antimicrob. Agents. Chemother.* **2010**, *54*, 2480–2488.
3. Chen, C. K.; Leung, S. S.; Guilbert, C.; Jacobson, M. P.; McKerrow, J. H.; Podust, L. M. *PLoS Negl. Trop. Dis.* **2010**, *4*, e651.
4. Sherman, W.; Day, T.; Jacobson, M. P.; Friesner, R. A.; Farid, R. *J. Med. Chem.* **2006**, *49*, 534–553.
5. Friesner, R. A.; Banks, J. L.; Murphy, R. B.; Halgren, T. A.; Klicic, J. J.; Mainz, D. T.; Repasky, M. P.; Knoll, E. H.; Shelley, M.; Perry, J. K.; Shaw, D. E.; Francis, P.; Shenkin, P. S. *J. Med. Chem.* **2004**, *47*, 1739–1749.
6. Zhu, K.; Shirts, M. R.; Friesner, R. A.; Jacobson, M. P. *J. Chem. Theory. Comput* **2007**, *3*, 640–648.
7. Huang, N.; Kalyanaraman, C.; Irwin, J. J.; Jacobson, M. P. *J. Chem. Inf. Model* **2006**, *46*, 243–253.
8. Morrison, J. F. *Biochim. Biophys. Act.a* **1969,** *185*, 269–286.

9. Engel, J. C.; Doyle, P. S.; Hsieh, I.; McKerrow, J. H. *J. Exp. Med.* **1998**, *188*, 725–734.

10. Engel, J. C.; Ang, K. K.; Chen, S.; Arkin, M. R.; McKerrow, J. H.; Doyle, P. S. *Antimicrob. Agents. Chemother*. **2010**, *54*, 3326–3334.

11. Engel, J. C.; Doyle, P. S.; Dvorak, J. A. *J. Protozool.* **1985**, *32*, 80–83.

12. McKerrow, J. H.; Doyle, P. S.; Engel, J. C.; Podust, L. M.; Robertson, S. A.; Ferreira, R.; Saxton, T.; Arkin, M.; Kerr, I. D.; Brinen, L. S.; Craik, C. S. *Mem. I. Oswaldo. Cruz.* **2009**, *104*, 263–269.

13. Rosenfeld, J.; Capdevielle, J.; Guillemot, J. C.; Ferrara, P. *Anal. Biochem.* **1992**, *203*, 173–179.

14. Bhattacharya, M.; Su, G.; Su, X.; Oses-Prieto, J. A.; Li, J. T.; Huang, X.; Hernandez, H.; Atakilit, A.; Burlingame, A. L.; Matthay, M. A.; Sheppard, D. Am *J. Physiol. Lung. Cell. Mol. Physiol.* **2012**, *303*, L12–L19.

15. Guan, S.; Price, J. C.; Prusiner, S. B.; Ghaemmaghami, S.; Burlingame, A. L. *Mol. Cell. Proteomics.* **2011**, *10.*

16. Jaishankar, P.; Hansell, E.; Zhao, D. M.; Doyle, P. S.; McKerrow, J. H.; Renslo, A. R. *Bioorg. Med. Chem. Lett.* **2008**, *18*, 624–628.

17. Speers, A. E.; Cravatt, B. F. *Chem. Biol.* **2004**, *11*, 535–546.

18. Yang, P.-Y.; Wang, M.; He, C. Y.; Yao, S. Q. *Chem. Commun.* **2012**, *48*, 835–837.

19. Somoza, J. R.; Zhan, H.; Bowman, K. K.; Yu, L.; Mortara, K. D.; Palmer, J. T.; Clark, J. M.; McGrath, M. E. *Biochemistry* **2000**, *39*, 12543–12551.
